# Epithelial Cell Mechanoresponse to Matrix Viscoelasticity and Confinement Within Micropatterned Viscoelastic Hydrogels

**DOI:** 10.1002/advs.202408635

**Published:** 2025-02-14

**Authors:** Giuseppe Ciccone, Mariana Azevedo Gonzalez‐Oliva, Marie Versaevel, Marco Cantini, Massimo Vassalli, Manuel Salmeron‐Sanchez, Sylvain Gabriele

**Affiliations:** ^1^ Institute for Bioengineering of Catalonia (IBEC) The Barcelona Institute for Science and Technology (BIST) Barcelona 08028 Spain; ^2^ Mechanobiology & Biomaterials Group University of Mons Research Institute for Biosciences CIRMAP, Place du Parc Mons 20 B‐7000 Belgium; ^3^ Centre for the Cellular Microenvironment University of Glasgow Advanced Research Centre 11 Chapel Lane Glasgow G11 6EW UK; ^4^ Institució Catalana de Recerca i Estudis Avançats (ICREA) Barcelona Spain

**Keywords:** confinement, epithelial cells, extracellular matrix, hydrogels, micropatterning, viscoelasticity

## Abstract

Extracellular matrix (ECM) viscoelasticity has emerged as a potent regulator of physiological and pathological processes, including cancer progression. Spatial confinement within the ECM is also known to influence cell behavior in these contexts. However, the interplay between matrix viscoelasticity and spatial confinement in driving epithelial cell mechanotransduction is not well understood, as it relies on experiments employing purely elastic hydrogels. This work presents an innovative approach to fabricate and micropattern viscoelastic polyacrylamide hydrogels with independently tuneable Young's modulus and stress relaxation, specifically designed to mimic the mechanical properties observed during breast tumor progression, transitioning from a soft dissipative tissue to a stiff elastic one. Using this platform, this work demonstrates that matrix viscoelasticity differentially modulates breast epithelial cell spreading, adhesion, YAP nuclear import and cell migration, depending on the initial stiffness of the matrix. Furthermore, by imposing spatial confinement through micropatterning, this work demonstrates that confinement alters cellular responses to viscoelasticity, including cell spreading, mechanotransduction and migration. These findings establish ECM viscoelasticity as a key regulator of epithelial cell mechanoresponse and highlight the critical role of spatial confinement in soft, dissipative ECMs, which was a previously unexplored aspect.

## Graphical abstract

Extracellular matrix (ECM) viscoelasticity and spatial confinement regulate cellular processes such as cancer progression, yet their interplay remains poorly understood. By developing fibronectin (FN)‐micropatterned viscoelastic hydrogels mimicking breast cancer mechanics, this study explores breast epithelial cell mechanoresponse to ECM viscoelasticity and examines how confinement alters this response, including spreading, adhesion, migration, and mechanotransduction. These findings offer new insights into cell mechanobiology in soft, confined, dissipative environments.

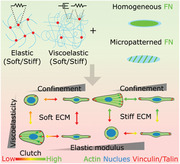

## 1 Introduction

The mechanical properties of the extracellular matrix (ECM) have emerged as crucial determinants of cellular behavior, and changes in ECM mechanics have been linked to disease progression.^[^ ^1^, ^2^ ^]^ Cells actively probe ECM mechanical properties through integrins and associated focal adhesions (FAs), forming a dynamic cross‐talk that profoundly influences cell spreading, migration, and differentiation.^[^ ^3^ ^]^

ECM mechanical properties have been predominantly replicated in terms of elasticity, commonly referred to as stiffness or rigidity. It is now widely acknowledged that matrix elasticity alone influences numerous pathophysiologically relevant cellular processes.^[^ ^4^ ^]^ For instance, recent studies have demonstrated that durotaxis, the process of migration along stiffness gradients,^[^ ^5^, ^6^, ^7^ ^]^ occurs in vivo in the *Xenopus laevis* neural crest, orchestrating the coordinated movement of cells.^[^ ^8^ ^]^

However, while much attention has been focused on matrix elasticity, ECM mechanics are intrinsically governed by viscoelasticity. Indeed, the mechanical response that cells probe is time dependent, with stress relaxation dictating the perceived mechanical properties that, in turn, drive cell behavior.^[^ ^9^ ^]^ The ECM is a complex polymeric network embedded in the extracellular fluid, rendering it a viscoelastic material.^[^ ^10^, ^11^ ^]^ More specifically, ECM viscoelasticity has been associated to the breaking of weak cross‐links, polymer entanglements, and protein unfolding,^[^ ^10^, ^11^ ^]^ resulting in the dissipation of stress exerted by cells over time.

While exploration of relevant relaxation times in vivo is still at its early stages,^[^ ^9^, ^12^ ^]^ the role of viscoelasticity in influencing cell responses in vitro has gained appreciation over the past decade. Notably, research has shown that matrix energy dissipation plays a regulatory role in fundamental processes such as cell spreading,^[^ ^13^, ^14^, ^15^ ^]^ differentiation,^[^ ^14^, ^16^, ^17^, ^18^ ^]^ and more recently cell migration^[^ ^19^, ^20^, ^21^ ^]^ and cancer progression.^[^ ^12^ ^]^ In the body, cell migration plays a crucial role in diverse processes, including development, wound healing, and cancer metastasis.^[^ ^22^ ^]^ The coordinated movement of cells is orchestrated by numerous biophysical factors, such as ECM stiffness^[^ ^5^, ^6^, ^7^ ^]^ and spatial confinement imposed by the ECM or neighboring cells.^[^ ^23^, ^24^, ^25^, ^26^, ^27^ ^]^ Of note, spatial confinement has been shown to influence migration decision‐making of cancer cells^[^ ^28^ ^]^ and is now considered as a potential regulator of metastasis.^[^ ^29^ ^]^

Cells in the body oftentimes migrate in confined spaces, such as along ECM fibres or through dense tissues, leveraging these guidance cues to facilitate migration along predetermined tracks, a phenomenon notably observed in cancer progression.^[^ ^24^ ^]^ Interestingly, it has been demonstrated that matrix confinement alters the relationship between cell migration speed and ECM stiffness.^[^ ^25^, ^30^, ^31^ ^]^ These results indicate a complex interplay between the mechanical properties of the matrix and the level of cellular confinement, both of which play a pivotal role in tumor invasiveness.^[^ ^24^, ^32^ ^]^ More recently, it has been reported that cancer tissue behaves as a viscoelastic material, exhibiting distinct viscoelastic properties that evolve alongside tumor progression.^[^ ^33^ ^]^ Specifically, breast cancer tissue consistently exhibits higher stiffness compared to surrounding healthy tissue,^[^ ^33^, ^34^, ^35^, ^36^, ^37^, ^38^, ^39^ ^]^ while being accompanied by a concomitant loss of viscoelasticity.^[^ ^33^, ^35^ ^]^ However, the exact relationship between matrix viscoelasticity and spatial confinement in driving epithelial cell mechanotransduction and migration during cancer progression remains unclear.

To tackle this challenge, we formulated a series of four polyacrylamide (PAAm) hydrogels with independently adjustable Young's modulus (*E*) and varying degrees of viscous dissipation, spanning the whole spectrum of mechanical properties observed during breast tumor progression.^[^ ^34^, ^35^, ^40^ ^]^ Although soft in absolute terms considering the stiffness range of biological tissues,^[^ ^1^, ^10^ ^]^ we denominated our hydrogels “soft” (*E* ≈ 0.3 kPa) and “stiff” (*E* ≈ 3 kPa) for consistency with recent literature^[^ ^40^ ^]^ and to highlight a 10‐fold relative stiffness change. We further classified the developed hydrogels as “elastic” (E) or “viscoelastic” (V) based on their stress relaxation profiles and loss tangent (tan(*δ*)) values, which were either lower or higher than 0.1, a value observed in most biological tissues.^[^ ^10^ ^]^ Critically, by employing soft micropatterning techniques to create one‐dimensional (1D) fibronectin (FN) lines on these viscoelastic hydrogels, we successfully investigated the interplay between spatial confinement and matrix viscoelasticity in the mechanoresponse of breast epithelial cells.^[^ ^23^, ^41^ ^]^ Our results shed light on the intricate interplay between elasticity, viscoelasticity and spatial confinement in orchestrating epithelial cell mechanobiology, offering insights into the role of time‐dependent matrix mechanics in the context of breast cancer progression.

## 2 Results and Discussion

### 2.1 Micropatterned Viscoelastic Polyacrylamide Hydrogels to Capture the Pathophysiology of the Breast Tissue Microenvironment

We engineered new PAAm hydrogels to emulate the viscoelastic properties characteristic of both healthy and cancerous breast tissue. Notably, PAAm hydrogels serve as a well‐established model in mechanobiology research owing to their adaptable and customizable physicochemical properties.^[^ ^42^, ^43^ ^]^ Differences in elasticity have been documented between normal and tumorous breast human tissues using different techniques, generally with values of *E* ≤ 1 kPa for non‐tumor tissue and *E* ≥ 3 kPa for malignant breast tumors.^[^ ^36^, ^37^ ^]^ Of note, using atomic force microscopy based nanoindentation, a mean *E ≈* 0.4 kPa for normal human breast tissue and *E* ≈ 3 kPa for invasive human cancer tissue was reported.^[^ ^38^ ^]^ Therefore, our initial focus was on fabricating standard linearly elastic hydrogels with *E* approximately of the above‐reported values, representing the stiffness of healthy and tumor breast tissues, respectively. Importantly, the relative stiffening observed via nanoindentation is consistent with bulk tissue stiffening observed using unconfined compression or shear rheology in a mouse model of breast tumor progression.^[^ ^39^ ^]^ Initial attempts in fabricating soft substrates (*E* ≈ 0.3 kPa) revealed the formation of surface defects during the detachment of hydrogel‐coverslip assemblies from the underlying glass support. This issue was likely due to the very low elastic modulus of the gels, as previously reported.^[^ ^44^, ^45^, ^46^ ^]^ Surface defects are known to influence cell behavior^[^ ^46^ ^]^ and would additionally make micropatterning impossible. To address this issue, we developed a novel methodology using a flexible, oxygen‐impermeable polychlorotrifluoroethylene (PCTFE) support. This approach allowed efficient free‐radical polymerization to occur, and ensured a straightforward and reproducible detachment process (Experimental Section) (Figure S1, Supporting Information). This new methodology enabled the formation of defect‐free PAAm hydrogels across a wide range of mechanical properties and was therefore used to develop all substrates in this work, including both elastic and viscoelastic matrices.

**Figure 1:**
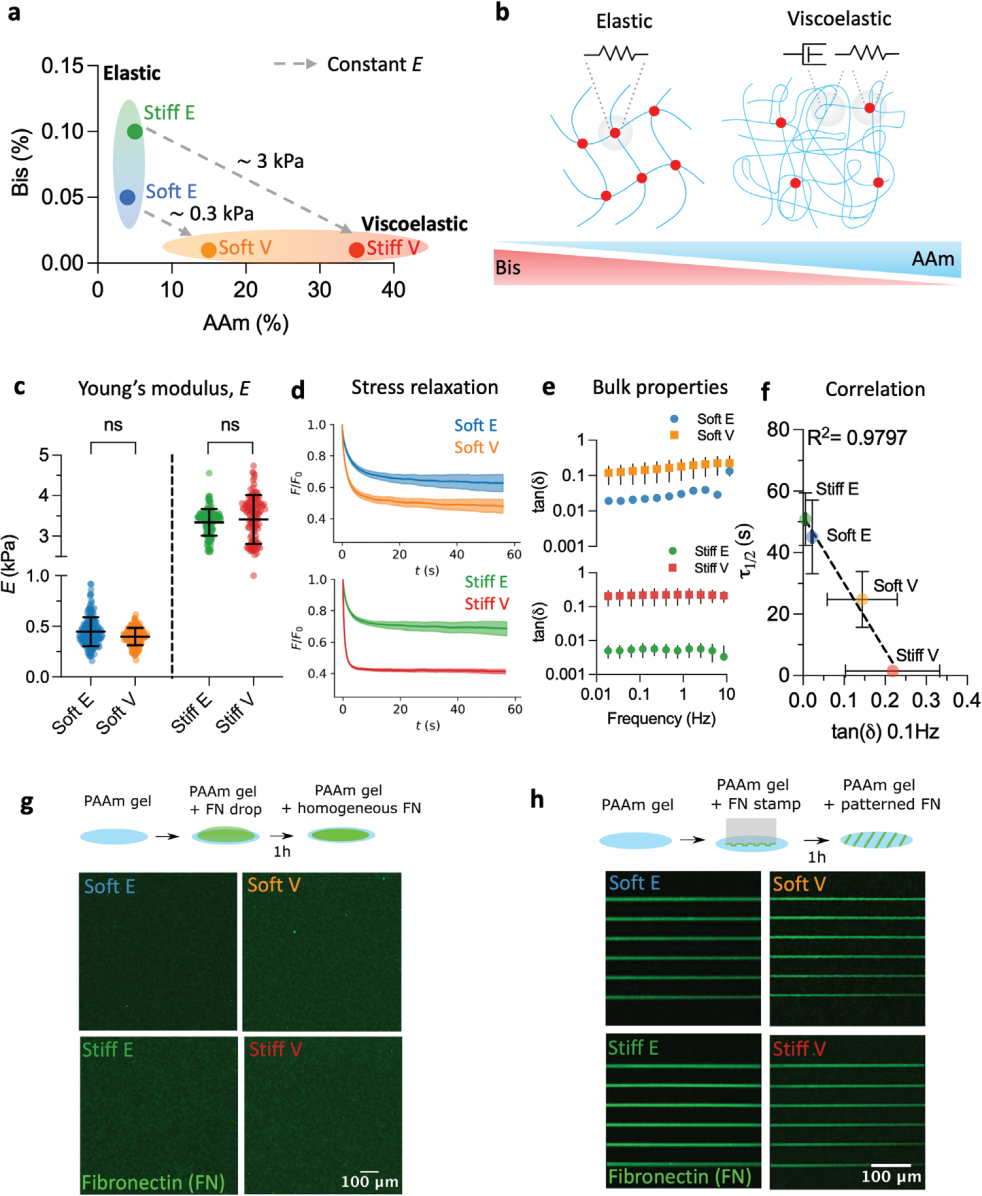
Micropatterned viscoelastic PAAm hydrogels to capture the pathophysiology of the breast tissue microenvironment. a) Phase diagram showing the percentages of bisacrylamide (Bis) cross‐linker and acrylamide (AAm) monomer used to tune the viscoelastic properties of PAAm hydrogels in this study. Viscoelastic hydrogels have a high AAm to Bis ratio compared to elastic hydrogels. Soft E = soft elastic, Soft V = soft viscoelastic, Stiff E = stiff elastic, Stiff V = stiff viscoelastic. The dashed gray lines connect hydrogels of similar Young's modulus (*E*) (≈ 0.3 and ≈ 3 kPa) but different viscoelastic properties. b) Schematic representation of the strategy used to obtain elastic and viscoelastic hydrogels with the same initial *E*. The amount of Bis is decreased while concurrently increasing the amount of AAm to favor physical entanglements. Red dots represent chemical cross‐links, idealized by an elastic spring. Chain entanglements are idealized by a viscous dashpot. c) *E* of hydrogels used in this work. Each point represents a single indentation, with at least 121 indentations (121≤ *n* ≤ 173) from three independently prepared samples. ns *p* = 0.3427 (Soft group) and *p* = 0.1453 (Stiff group), two‐way ANOVA with Bonferroni's multiple comparisons test. Data is shown as mean ± SD. d) Average stress relaxation profiles of hydrogels used in this work. Curves were obtained by averaging at least 121 individual curves (121≤ *n* ≤ 151) coming from at least two independent experiments. Data is shown as mean ± SD. e) tan(*δ*) obtained from bulk rheology oscillatory sweeps of hydrogels used in this work (strain 1 %, Experimental Section). Data has been averaged over three independent samples. Data is shown as mean ± SD. f) Correlation between the relaxation half‐time ($\tau_{\frac{1}{2}})$ obtained from nanoindentation experiments and the tan(*δ*) at 0.1 Hz obtained from bulk rheology experiments for the same data shown in d and e (*R* ^2^ = 0.9797, mean ± SD). Note that elastic hydrogels dissipated less than 50% of the original stress, so the relaxation half time was taken from the time point resulting in a stress value as close as possible to 50%. g) Representative images of homogeneous fibronectin (FN) coating on elastic and viscoelastic PAAm hydrogels. h) Representative images of micropatterned FN coating on elastic and viscoelastic PAAm hydrogels.

Elastic hydrogels with *E* of ≈ 0.3 kPa and ≈ 3 kPa were developed by maintaining the acrylamide (AAm) monomer concentration at ≈ 4% and increasing the bisacrylamide (Bis) cross‐linker concentration from ≈ 0.05% to ≈ 0.1%. Quasi‐static nanoindentation experiments performed using a commercially available fibre‐optic based nanoindentation technique (Experimental Section) confirmed these mechanical properties. We denoted these hydrogels “soft elastic” and “stiff elastic” (Soft E/Stiff E), respectively (**Figure** **1**a,c).

Our next objective was to formulate hydrogels with nearly identical *E* but enhanced stress relaxation, aiming to dissect the influence of energy dissipation on cellular behavior. Various strategies have been proposed to independently control the stiffness and viscoelasticity of PAAm hydrogels, including integrating viscous pre‐polymerized linear AAm into an elastic network,^[^ ^14^ ^]^ adjusting the initiator and activator concentrations,^[^ ^20^ ^]^ or modifying the AAm‐to‐Bis ratio.^[^ ^16^, ^18^ ^]^ In the latter approach, viscoelasticity is enhanced by increasing the concentration of monomer while concurrently decreasing the concentration of cross‐linker (Figure 1b). In this scenario, the network's elasticity is governed by the monomer concentration, while the low concentration of cross‐linker favors physical entanglements between long and loosely bound PAAm chains, providing viscoelastic characteristics to the material.^[^ ^16^, ^18^ ^]^ By maintaining the Bis concentration at ≈ 0.01% and increasing the AAm concentration from 15% to 35%, we produced viscoelastic PAAm hydrogels with initial *E* akin to that of elastic hydrogels (Figure 1a,c). We termed these gels “soft viscoelastic” and “stiff viscoelastic” (Soft V/Stiff V). As previously introduced, we opted for this terminology (i.e., soft versus stiff) for consistency with recent literature.^[^ ^40^ ^]^ However, it is important to note that the hydrogels used in this study are soft in absolute terms.^[^ ^1^, ^10^ ^]^ Hence, the distinction between “soft” and “stiff” more accurately reflects a 10‐fold relative change in *E* between the two hydrogel groups.

To confirm the augmented dissipative properties of the viscoelastic hydrogels, we conducted nanoindentation stressrelaxation experiments using the same fibre‐optic nanoindentation platform as well as bulk rheology oscillatory analyses (Experimental Section). Importantly, using similar nanoindentation techniques, it has been shown that mouse breast cancer tissue shows decreased stress relaxation compared to healthy tissue and the surrounding stroma.^[^ ^35^ ^]^ Nanoindentation stressrelaxation experiments, aimed at assessing viscoelasticity at similar force and length scales experienced by cells,^[^ ^47^, ^48^, ^49^ ^]^ entailed applying a step indentation of ≈ 10% of the bead's radius for 60 s, resulting in a physiologically relevant 2D strain of approximately 7% (Experimental Section).^[^ ^17^ ^]^ Viscoelastic hydrogels demonstrated enhanced and faster stress relaxation compared to their elastic counterparts (Figure 1d), with viscoelastic hydrogels dissipating more than 50% of the initial force during the experiment (Figure S2a,b, Supporting Information). All hydrogels reached a plateau in stress relaxation within 60 s, with a fraction of the stress remaining undissipated. Notably, our hydrogels exhibited behavior characteristic of viscoelastic solids, rather than fluids, across the physiologically relevant mechanosensitive time scale under investigation, experimentally estimated to be over tens of seconds from traction force fluctuations.^[^ ^14^ ^]^ It is important to note that in an ideal elastic solid, there is no energy dissipation, and therefore the stress remains constant over time under the applied strain. As a result, the stress relaxation half‐time ($\tau_{\frac{1}{2}}$, that is, the time taken for the stress to relax to half of its initial value) is considered infinite in ideal elastic materials. Importantly, the relaxation dynamics and associated time scales exhibit significant variability depending on the measurement scale, spanning from bulk measurements (e.g., through bulk compression tests) to local ones (e.g., via nanoindentation).^[^ ^11^, ^47^ ^]^ As a result, the diversity in measurement techniques presents considerable obstacles to directly compare relaxation timescales of hydrogels across different studies.

We further confirmed the viscoelastic properties of the bulk hydrogels through oscillatory bulk rheology tests (Figure 1e, and Figure S3 and Note S1, Supporting Information). Specifically, we assessed the ratio between the loss modulus and the storage modulus commonly referred to as the loss tangent or tan(*δ*), which serves as an indicator of material viscosity.^[^ ^11^ ^]^ Interestingly, it has been shown that viscoelastic ECMs exhibit high tan(*δ*) values, typically exceeding 0.1.^[^ ^10^, ^11^ ^]^ Our analyses revealed consistently higher tan(*δ*) values for viscoelastic hydrogels compared to their elastic counterparts across the entire frequency range tested (Figure 1e), thus confirming their enhanced dissipative properties.

Correlation analysis between $\tau_{\frac{1}{2}}$ obtained from nanoindentation experiments and the tan(*δ*) at 0.1 Hz obtained from bulk oscillatory experiments revealed a robust linear relationship between these variables (Figure 1f, *R* ^2^ = 0.9797). This suggests that both metrics serve as reliable indicators of the viscoelastic behavior of the hydrogels developed in this study. We selected 0.1 Hz as it corresponds to a similar equivalent timescale as the one in stress relaxation experiments (tens of seconds), rendering it a biologically relevant frequency, as previously proposed.^[^ ^14^ ^]^

We further observed a good correlation between the stress relaxation amplitude (i.e., the percentage of energy dissipated over the experiment's time) and the tan(*δ*) at 0.1 Hz, corroborating the enhanced dissipative properties of viscoelastic hydrogels compared to their elastic counterparts (Figure S2c, Supporting Information).

Given the nonadhesive nature of PAAm hydrogels, functionalization with ECM proteins is essential to allow for cell adhesion.^[^ ^42^, ^50^ ^]^ Conventional PAAm gel functionalization typically involves UV activation of the heterobifunctional cross‐linker Sulfo‐SANPAH.^[^ ^42^ ^]^ However, the compound's high instability in aqueous solutions often leads to substantial variability in protein cross‐linking efficiency on the hydrogel surface.^[^ ^45^, ^51^ ^]^ This instability poses a significant challenge, particularly for micropatterning hydrogels, where the time required to dry the hydrogel surface exceeds the compound's half‐life.^[^ ^51^ ^]^ To address this challenge, we enabled binding of ECM proteins to PAAm hydrogels through the incorporation of reactive aldehyde groups within the network.^[^ ^51^, ^52^ ^]^ This key modification facilitates the covalent conjugation of EMC proteins onto the hydrogel surface regardless of the hydrogel mechanical properties, including viscoelastic substrates, removing the need for additional activation steps post‐gelation.^[^ ^51^, ^52^ ^]^ FN, an important component of the interstitial breast ECM whose upregulation is associated to breast cancer progression,^[^ ^53^ ^]^ was conjugated onto the hydrogels either homogeneously or through micropatterning narrow lines of approximately 5 µm in width via microcontact printing (Experimental Section) (Figure 1g,h). Fluorescently labelling FN and imaging the surface of the hydrogels confirmed no significant changes in FN intensity in either case, ensuring a consistent amount of FN availability irrespective of the underlying matrix viscoelastic properties (Figure S4, Supporting Information).

In summary, we have established a robust system that faithfully reproduces the entire spectrum of mechanical properties observed during breast cancer progression, specifically the transition from a soft viscoelastic tissue to a stiff elastic one.^[^ ^35^, ^38^ ^]^ Notably, our innovative methodology for fabricating soft and stress relaxing PAAm hydrogels (Figure S1, Supporting Information), enabled the introduction of FN micropatterns on these substrates. This advancement allowed us to investigate the effects of elasticity, viscoelasticity, and spatial confinement on cell behavior, while retaining precise control over experimental conditions within a previously unexplored regime of physicochemical properties.

### 2.2 Viscoelasticity Modulates Cell Spreading, Focal Adhesions, and YAP Nuclear Import in Opposite Directions on Soft and Stiff Substrates

We first investigated the impact of matrix viscoelasticity on key cellular processes, including cell spreading, adhesion formation and nuclear YAP translocation. Previous studies have shown that cell responses to viscoelasticity are cell‐type dependent and heterogeneous.^[^ ^54^, ^55^ ^]^ Additionally, it is unknown how stress relaxation affects cell mechanobiology in extremely soft matrices (*E *< 1 kPa), primarily due to technical challenges in reliably fabricating such substrates. Yet, soft tissues are the ones exhibiting the most pronounced dissipative properties.^[^ ^10^, ^11^ ^]^

To delineate epithelial cell response to matrix energy dissipation, we employed MCF‐10A cells, a well‐established model for the breast epithelium.^[^ ^56^, ^57^ ^]^ By focussing on single cells, we eliminated confounding effects introduced by cell–cell interactions, thereby isolating ECM mechanics‐mediated mechanotransduction effects.^[^ ^56^, ^58^ ^]^ Although cell–cell contacts are fundamental in epithelial tissues, this approach has proven useful to study epithelial cell mechanoresponse to emerging properties such as matrix viscoelasticity,^[^ ^13^, ^21^ ^]^ allowing to simplify the experimental system to its minimal components.

**Figure 2:**
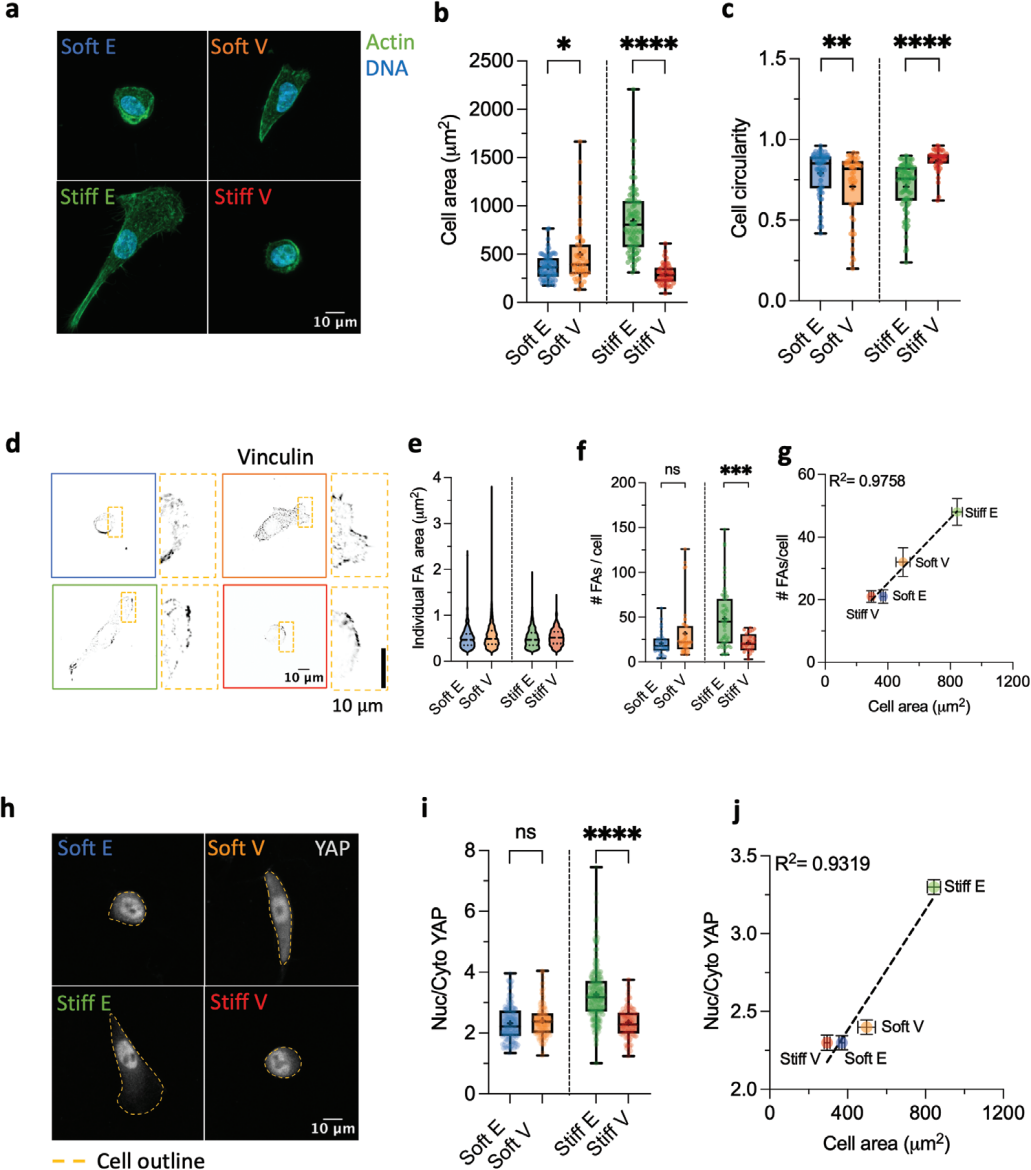
Viscoelasticity modulates cell spreading, FAs, and YAP nuclear import in opposite directions on soft and stiff substrates. a) Representative Actin/DNA images of typical MCF‐10A cell morphologies observed on elastic and viscoelastic PAAm matrices. b) Quantification of MCF‐10A cell spreading area on Soft E (*n* = 74 cells), Soft V (*n* = 51 cells), Stiff E (*n* = 100 cells), and Stiff V (*n* = 49 cells) hydrogels from at least two independent experiments. ^*^ *p* = 0.0148, ^****^ *p* < 0.0001, two‐way ANOVA with Bonferroni's multiple comparisons test. c) Quantification of MCF‐10A cell circularity on Soft E (*n =* 74 cells), Soft V (*n =* 51 cells), Stiff E (*n =* 100 cells), and Stiff V (*n =* 49 cells) hydrogels from at least two independent experiments. ^**^ *p* = 0.0061, ^****^ *p* < 0.0001, two‐way ANOVA with Bonferroni's multiple comparisons test. d) Representative FAs (Vinculin) images of MCF‐10A cells cultured on elastic and viscoelastic PAAm hydrogels. e) Distribution of individual FA area of MCF‐10A cells cultured on Soft E (*n* = 685 adhesions), Soft V (*n* = 1301 adhesions), Stiff E (*n* = 3055 adhesions) and Stiff V (*n* = 580 adhesions) hydrogels. Data was obtained from at least two independent experiments. f) Quantification of the number of FAs per cell (#FAs/cell) of MCF‐10A cells cultured on Soft E (*n* = 33 cells), Soft V (*n* = 41 cells), Stiff E (*n* = 58 cells) and Stiff V (*n* = 27 cells) hydrogels from at least two independent experiments. ns *p* = 0.1413, ^****^ *p* < 0.0001, two‐way ANOVA with Bonferroni's multiple comparisons test. g) Plotting the #FAs/cell versus the cell spreading area reveals a linear relationship between the two variables (*R* ^2^ = 0.9758, mean ± SEM). h) Representative YAP images of MCF‐10A cells cultured on elastic and viscoelastic PAAm hydrogels. The cell's outline is highlighted by a dashed yellow line. Note the absence of almost any cytoplasmic YAP on Stiff E matrices compared to the other conditions. i) Quantification of the Nuclear to Cytoplasmic (Nuc/Cyto) YAP ratio of MCF‐10A cells cultured on Soft E (*n* = 157 cells), Soft V (*n* = 128 cells), Stiff E (*n* = 320 cells), Stiff V (*n* = 113 cells) hydrogels from at least two independent experiments. ns *p* = 0.5161, ^****^ *p* < 0.0001, two‐way ANOVA with Bonferroni's multiple comparisons test. j) Nuc/Cyto YAP ratio increases linearly with cell spreading area on viscoelastic PAAm hydrogels (*R* ^2^ = 0.9319, mean ± SEM).

After culturing cells on viscoelastic PAAm gels for at least 24 h, we observed significant differences in cell spreading area and morphology as a function of substrate viscoelastic properties, as quantified by immunofluorescence (**Figure** **2**a–c). Notably, cell spreading area increased with enhanced viscoelasticity on soft hydrogels and decreased with increased viscoelasticity on stiff hydrogels (Figure 2a,b). Overall, cell spreading area was minimal on Stiff V and Soft E hydrogels, maximal on Stiff E hydrogels, and intermediate on Soft V hydrogels. Cell circularity exhibited an opposite trend to cell spreading area (Figure 2b,c), suggesting a reciprocal relationship. To further validate that mammary epithelial cells perceive Stiff E hydrogels as substrates with high stiffness, we cultured MCF‐10A cells on FN‐coated glass coverslips. Interestingly, we observed no significant difference in cell spreading area and cell circularity compared to cells cultured on Stiff E hydrogels (Figure S5, Supporting Information), confirming that breast epithelial cells perceive Stiff E hydrogels as substrates exhibiting infinite rigidity in terms of spreading area and morphology.

While increased matrix rigidity is traditionally known to promotes cell spreading,^[^ ^4^ ^]^ conflicting results have been reported regarding the effects of increased viscoelasticity on cell spreading. Previous studies suggested either inhibition or enhancement of cell spreading with increased matrix viscoelasticity, partially due to confounding effects introduced by viscoplasticity.^[^ ^13^, ^14^, ^15^, ^54^, ^59^, ^60^ ^]^ Our findings demonstrate that increased viscoelasticity distinctly modulates cell spreading, enhancing it when the initial substrate stiffness is low (*E* ≤ 1 kPa) and suppressing it when the initial substrate stiffness is high (*E* ≥ 1 kPa).^[^ ^13^, ^59^ ^]^ Critically, since PAAm hydrogels are chemically cross‐linked, these effects are associated with viscoelasticity alone.^[^ ^61^ ^]^ Interestingly, our results align with previous theoretical predictions using molecular clutches interacting with a viscoelastic substrate. These models suggest a regime of physicochemical properties represented by a combination of low stiffness and high ligand density in which cell spreading is enhanced compared to a purely elastic matrix of the same stiffness.^[^ ^13^, ^59^ ^]^ To validate the role of ligand density within our experimental system, we reduced the FN concentration utilized for functionalizing PAAm hydrogels by sevenfold, from 70 to 10 µg mL^−1^. This reduction abrogated the previously observed increase in cell spreading on Soft V matrices compared to Soft E ones, while still preserving the diminished spreading observed on Stiff V matrices relative to Stiff E matrices (Figure S6, Supporting Information), again consistent with previous theoretical simulations.^[^ ^13^, ^59^ ^]^ Importantly, the use of FN in our system ensures that the observed effects are due to viscoelasticity‐mediated mechanotransduction, rather than ligand tethering effects, a phenomenon specific to collagen I that can result in fibres formation on the surface of PAAm hydrogels.^[^ ^14^, ^62^, ^63^ ^]^

Cell spreading in 2D serves as a proxy for mechanically driven increased cytoskeletal tension and cell contractility.^[^ ^64^ ^]^ Focusing on vinculin, a FA protein within the molecular clutch axis,^[^ ^59^, ^65^, ^66^ ^]^ we found no biologically meaningful variations of individual FA area across the different matrices (Figure 2d,e), suggesting that FA reinforcement does not occur over the range of ECM viscoelastic properties explored.^[^ ^67^ ^]^ However, our findings revealed that the number of FAs per cell increased linearly with the projected cell spreading area (Figure 2f,g, *R* ^2^ = 0.9758). These results demonstrate that increased matrix viscoelasticity enhances cell contractility at low initial stiffness, while conversely diminishing cell contractility when the initial stiffness of the ECM is elevated.^[^ ^64^ ^]^ Meanwhile, FA density remained almost constant, suggesting that elevated matrix viscoelasticity supports cell adhesion within the entire range of explored mechanical properties.

On 2D ECMs, the mechanical tension of actin stress fibres acts on the nucleus through the Linker of Nucleoskeleton and Cytoskeleton (LINC) complex, facilitating YAP nuclear import—a fundamental transcriptional regulator mediating mechanotransduction.^[^ ^68^ ^]^ Interestingly, we observed predominantly nuclear YAP in all conditions, with Nuclear (Nuc)/Cytoplasmic (Cyto) ratios exceeding 2 (Figure 2h,i). We hypothesized that the heightened FN concentration utilized in this work likely contributed to the elevated basal level of nuclear YAP across all conditions, a phenomenon recognized to enhance nuclear YAP irrespective of ECM mechanical properties.^[^ ^69^ ^]^ To confirm this, we cultured cells on the same viscoelastic hydrogels but employed a sevenfold lower FN concentration, from 70 to 10 µg mL^−1^, resulting in the abolishment of YAP nuclear localization across all conditions except for Stiff E matrices, as anticipated (Figure S7, Supporting Information). Nevertheless, nuclear YAP import remained responsive to ECM viscoelastic properties even at high FN ligand density, exhibiting a linear increase with cell projected area (Figure 2j, *R* ^2^ = 0.9319) and increasing with the number of FAs per cell (Figure S8, Supporting Information). These results underscore that cellular contractility governs nuclear YAP import in response to matrix viscoelastic properties.

Altogether, our findings reveal that matrix viscoelasticity, alongside stiffness, is as a key mediator of mechanotransduction in ECMs that mimic both physiological and pathological conditions in mammary epithelial cells. Importantly, we demonstrate that stress relaxation alone is sufficient to enhance cell spreading, adhesion formation and nuclear YAP translocation in extremely soft matrices (*E ≈* 0.3 kPa, i.e., equivalent to a shear modulus of 100 Pa). In contrast, stress relaxation suppresses the same processes in stiff (*E* ≈ 3 kPa, i.e., equivalent to a shear modulus of 1 kPa) matrices. These results highlight the pivotal role of stress relaxation within soft tissues in the regulation of their mechanoresponse, offering new insights into the complex interplay between matrix mechanical properties and cellular behavior.

### 2.3 Viscoelasticity Enhances Migration Speed and Persistence on Soft Substrates, While Impeding Them on Stiff Substrates via Actin Retrograde Flow and Adhesions Regulation

Acknowledging the pivotal role of FAs as critical mediators of cell migration through molecular clutch mechanisms,^[^ ^70^ ^]^ we next sought to investigate the influence of ECM viscoelasticity on cell migration. Recent studies have indicated that matrix stress relaxation enhances MCF‐10A migration—as well as other epithelial cells—on relatively soft fast‐relaxing alginate‐reconstituted basement membrane (rBM) hydrogels (*E* = 2 kPa) compared to their slow‐relaxing counterparts.^[^ ^21^ ^]^ While these findings are significant for understanding viscoelastic matrices resembling cancerous breast tissues, questions persist regarding how heightened energy dissipation impacts epithelial cell migration within mechanical microenvironments resembling the low stiffness of healthy (*E* ≈ 0.3 kPa) and high stiffness of cancerous (*E* ≈ 3 kPa) breast tissue.^[^ ^34^, ^35^, ^36^, ^37^, ^38^, ^39^, ^40^ ^]^ Furthermore, recent demonstrations of the evolution of tissue viscoelastic properties during breast tumor progression^[^ ^33^, ^34^, ^35^ ^]^ has fueled our interest in elucidating the role of matrix viscoelasticity in driving breast epithelial cell migration on soft and stiff elastic and viscoelastic ECMs.

**Figure 3:**
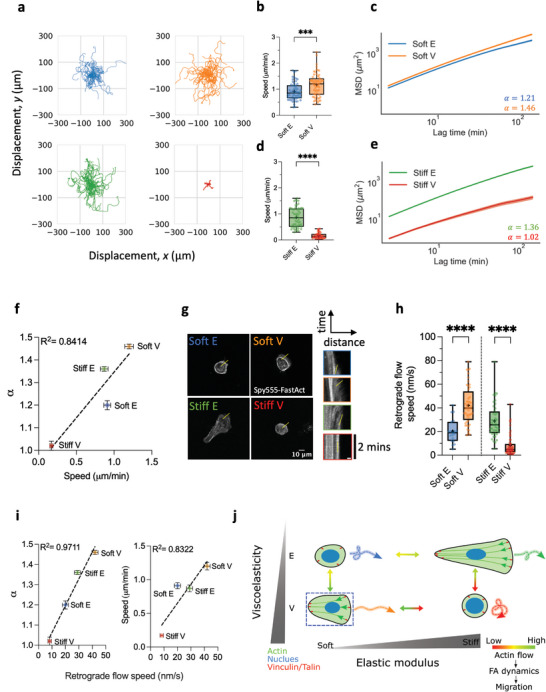
Viscoelasticity enhances migration speed and persistence on soft substrates, while impeding them on stiff substrates via actin retrograde flow and adhesions regulation. a) Representative *x–y* trajectories of MCF‐10A cells on elastic and viscoelastic PAAm hydrogels over 5 h (*n* = 48 trajectories for Soft E, *n* = 43 trajectories for Soft V, *n* = 49 trajectories for Stiff E, *n* = 34 trajectories for Stiff V). b) MCF‐10A cell migration speed on soft elastic (Soft E, *n* = 56 cells) and viscoelastic (Soft V, *n* = 61 cells) matrices obtained from three independent experiments. ^***^ *p* = 0.0007, unpaired two‐tailed *t*‐test. c) Average mean square displacement (MSD) versus lag time for MCF‐10A cells on soft elastic (Soft E) and viscoelastic (Soft V) matrices. The diffusion exponent, *α*, is shown in the graph. Data is shown as mean ± SEM (*n* = 48 cells for Soft E, *n* = 43 cells for Soft V) from three independent experiments. d) MCF‐10A cell migration speed on stiff elastic (Stiff E, *n* = 55 cells) and viscoelastic (Stiff V, *n* = 35 cells) matrices obtained from at least two independent experiments. ^***^ *p* = 0.0007, unpaired two‐tailed *t*‐test. ^****^ *p* < 0.0001, unpaired two‐tailed *t*‐test. e) Average MSD versus lag time for cells on stiff elastic (Stiff E) and viscoelastic (Stiff V) matrices. The diffusion exponent, *α*, is shown in the graph. Data is shown as mean ± SEM (*n* = 49 cells for Stiff E, *n* = 34 cells for Stiff V) from at least two independent experiments. f) Diffusion exponent, *α*, plotted against average cell migration speed (*R* ^2^ = 0.8414, mean ± SEM) for the same number of cells and independent experiments as in b–d (for cell migration speed) and c–e (for MSD). g) Representative images of MCF‐10A tagged with live Spy555‐FastAct on elastic and viscoelastic PAAm hydrogels. Yellow line shows location where kymographs were computed, on average. Insets show representative kymographs for each condition, with yellow line indicating the slope from which the actin retrograde flow speed is computed. The spatial scale bar in the inset is 2 µm, whereas the temporal scale bar is 2 min. h) Quantification of actin retrograde flow speed for MCF‐10A cells cultured on Soft E (*n* = 19 kymographs), Soft V (*n* = 48 kymographs), Stiff E (*n* = 44 kymographs) and Stiff V (*n* = 52 kymographs) hydrogels from at least two independent experiments. ^****^ *p* < 0.0001, two‐way ANOVA with Bonferroni's multiple comparisons test. i) Diffusion exponent, *α*, versus actin retrograde flow speed (left, *R* ^2^ = 0.9711); and migration speed versus actin retrograde flow speed (right, *R* ^2^ = 0.8322). Data is shown as mean ± SEM for the same number of cells/kymographs as in c‐e and h, respectively. j) Schematic summary of experimental findings on how matrix viscoelasticity modulates MCF‐10A cell migration speed and persistence via actin retrograde flow speed and adhesion dynamics. Green arrows inside the cells represent actin retrograde flow, whereas colorful arrows outside the cell depict schematic migration trajectories. Dashed blue box shows condition where optimal migration occurs (Soft V).

We conducted 15‐h time lapse experiments to study the migratory behavior of MCF‐10A cells on elastic and viscoelastic PAAm hydrogels. By tracking individual cells, we quantified their average speed and mean square displacement (MSD).^[^ ^71^ ^]^ Migration tracks in **Figure** **3**a reveal that enhanced energy dissipation has a dual effect on cell migration (Movies S1–S4, Supporting Information). Specifically, cells on Soft V substrates migrated faster and further than cells on their elastic counterparts (i.e., Soft E), exhibiting both increased average speed and MSD (Figure 3b,c). Conversely, both speed and MSD significantly decreased for cells on Stiff V matrices compared to cells on Stiff E ones (Figure 3d,e). The slope of the MSD‐lag time data yielded the diffusion exponent, *α*, indicating migration persistence.^[^ ^71^ ^]^ Cells on Soft V substrates displayed higher persistence than those on Soft E substrates, while cells on Stiff E substrates were more persistent than cells on Stiff V ones. Additionally, we observed no significant difference in migration speed between cells cultured on Soft V substrates compared to those cultured on FN‐coated glass coverslips (Figure S9a, Supporting Information). However, cells on Soft V substrates had a diffusion exponent greater than 1 (*α* = 1.46) whereas cells on glass displayed sub‐diffusive migration (*α *= 0.82) (Figure S9b,c, Supporting Information). This confirms that enhanced viscoelasticity on soft substrates promotes cell migration to greater levels compared to infinitely stiff elastic substrates.

Overall, our results reveal that enhanced ECM viscoelasticity can either promote or hinder 2D cell migration depending on initial ECM stiffness. Previous reports suggested either an enhancement^[^ ^21^ ^]^ or abrogation^[^ ^19^, ^20^ ^]^ of cell migration in response to increased viscoelasticity, but these studies were limited to a single substrate stiffness. In addition, different cell types have varying number of active molecular motors and clutches that have been shown to establish a stiffness optimum for cell migration.^[^ ^7^ ^]^ Consequently, matrix viscoelasticity is likely to affect cell migration in nontrivial ways, depending on the initial stiffness of the substrate, relaxation time scale, and ECM ligand type and density.^[^ ^55^ ^]^

Furthermore, we observed a strong linear correlation between cell persistence and cell speed (Figure 3f, *R* ^2^ = 0.8414), a universal characteristic of migrating cells in vitro and in vivo regardless of matrix dimensionality (i.e., 2D versus 3D) and migration mode.^[^ ^72^ ^]^ This relationship is governed by actin polymerization rate, indicating that actin flow speed links the observed universal coupling between cell speed and persistence.^[^ ^72^ ^]^ To delve into the mechanism, we measured actin flow speed at the lamellipodium edge of migrating cells. Cells migrating on Stiff E matrices displayed a fan‐shaped phenotype with an extended lamellipodium, while cells on Soft V matrices exhibited a more compacted morphology with a short lamellipodium. Cells on Soft E hydrogels did not develop a prominent lamellipodium, similar to those on Stiff V hydrogels (Figure S10 and Movies S5–S8, Supporting Information). MCF‐10A cells were tagged to label fast‐polymerizing actin filaments (Experimental Section), and cells were imaged at high spatial and temporal resolution for 2 min at 1‐s intervals (Figure 3g and Movies S9–S12, Supporting Information). Using kymographs, we quantified the actin retrograde flow speed at the lamellipodium edge for cells on Soft V and Stiff E matrices, and at random anterior locations for cells on Soft E and Stiff V matrices (Figure 3h). Our findings indicated that the retrograde flow speed increased twofold by adding dissipative components in soft matrices, while in contrary it was impeded by incorporating viscous dissipation in stiff matrices (Figure 3h). Interestingly, we observed a robust linear correlation between the diffusion exponent and the actin retrograde flow speed (Figure 3i, *R* ^2^ = 0.9711), as well as the migration speed and the actin retrograde flow speed (Figure 3i, *R* ^2^ = 0.8322), indicating that the viscoelastic properties of the cell substrate regulate cell persistence and speed through modulation of the retrograde flow of actin filaments.^[^ ^73^ ^]^ These results suggest that maximal retrograde flow occurs on Soft V substrates (*E ≈* 0.3 kPa). Conversely, on Stiff V substrates (*E* = 3 kPa), viscosity significantly reduces cell spreading (Figure 2b), cell speed (Figure 3d), cell persistence (Figure 3e) and retrograde flow speed (Figure 3h) by impeding the backward movement of actin filaments. A reduction in actin flow speed is typically associated with enhanced cell spreading and mechanoactivation, as predicted by the molecular clutch model.^[^ ^59^, ^65^ ^]^ However, the cell region where actin flow is measured significantly determines its speed.^[^ ^74^ ^]^ Here, we measured the actin flow at the very edge of the cell lamellipodium (where present), which is associated with the highest actin flow in migrating cells.^[^ ^73^, ^75^ ^]^ Therefore, we attribute this discrepancy to where the actin flow is measured. Overall, our results are consistent with a polarity cue model which regulates cell migration via actin polymerization.^[^ ^72^ ^]^ To further investigate these processes, we transfected MCF‐10A cells with talin‐GFP to visualize FA dynamics. FAs were imaged over a 30‐min period (Movies S13–S16, Supporting Information). Migrating cells on Soft V and Stiff E substrates displayed highly dynamic adhesions, whereas adhesions were less dynamic on Soft E substrates and stationery on Stiff V substrates. These observations are consistent with the notion that FA formation and disassembly, coupled with actin retrograde flow, are complementary and essential mechanisms in the regulation of cell migration.^[^ ^70^ ^]^

Taken together, our results underscore the critical role of substrate viscoelasticity in modulating epithelial cell migration, revealing distinct responses dependent on substrate stiffness (Figure 3j). Notably, we observed that shorter stress relaxation times enhance cell migration on soft substrates (*E ≈* 0.3 kPa) while significantly impeding it on stiff substrates (*E* ≈ 3 kPa). At a first sight, our findings may seem to contradict recent results obtained on 2 kPa viscoelastic alginate‐rBM hydrogels.^[^ ^21^ ^]^ However, a closer comparison reveals consistency. Specifically, when epithelial cell migration on 2 kPa slow‐relaxing alginate‐rBM hydrogels was compared to migration on elastic PAAm hydrogels of the same elastic modulus, the authors found that both cell speed and directional persistence were higher on purely elastic PAAm hydrogels compared to slow‐relaxing alginate‐rBM ones.^[^ ^21^ ^]^ Since the viscoelastic hydrogels developed in our work are chemically cross‐linked, their bulk relaxation half time is expected to be within the range of several hundred to a few thousand of seconds,^[^ ^11^ ^]^ comparable to slow‐relaxing hydrogels reported previously.^[^ ^21^ ^]^ Consequently, our findings align with recent results demonstrating the effects of substrate stress relaxation on cell migration on matrices with elastic moduli in the range of 2–3 kPa.

However, our findings also reveal an intriguing regime on softer substrates (*E* ≈ 0.3 kPa), wherein mammary epithelial cells exhibit optimal migration on Soft V substrates, which does not occur when substrates are purely elastic.^[^ ^56^ ^]^ This is consistent with how stress relaxation is sufficient to promote mechanoactivation (spreading, adhesions and YAP translocation) on soft matrices. Drawing upon the evolving understanding of tissue viscoelastic properties during breast tumor progression,^[^ ^33^, ^34^, ^35^ ^]^ these results underscore the pivotal role of substrate viscoelasticity as a fundamental ECM physical property, profoundly influencing the regulation of cell migration across different stiffness regimes via regulation of actin and FA dynamics (Figure 3j).

### 2.4 Spatial Confinement Blunts Viscoelasticity‐Mediated Effects on Soft Matrices and Reduces Them on Stiff Matrices

In addition to changes in viscoelasticity within their microenvironment, epithelial mammary cells encounter spatial constraints imposed by neighboring cells or the ECM.^[^ ^24^ ^]^ Confined spaces have been shown to significantly influence cell migration,^[^ ^25^, ^26^, ^27^, ^30^, ^31^, ^76^ ^]^ with spatial confinement playing a crucial role in metastatic spreading during cancer progression.^[^ ^24^ ^]^ Interestingly, 1D confinement has been demonstrated to induce a spindle‐like morphology and migration phenotype resembling that of cells in soft 3D matrices,^[^ ^23^ ^]^ effectively capturing certain aspects of 3D cell migration.^[^ ^77^ ^]^ While few previous studies have explored the interplay between substrate stiffness and confinement in terms of cell migration,^[^ ^25^, ^30^, ^31^ ^]^ the impact of the coupling between increased matrix viscoelasticity and spatial confinement on cell migration remains elusive, mainly due to lack of experimental platforms allowing for the isolation of each variable on cell behavior.

**Figure 4:**
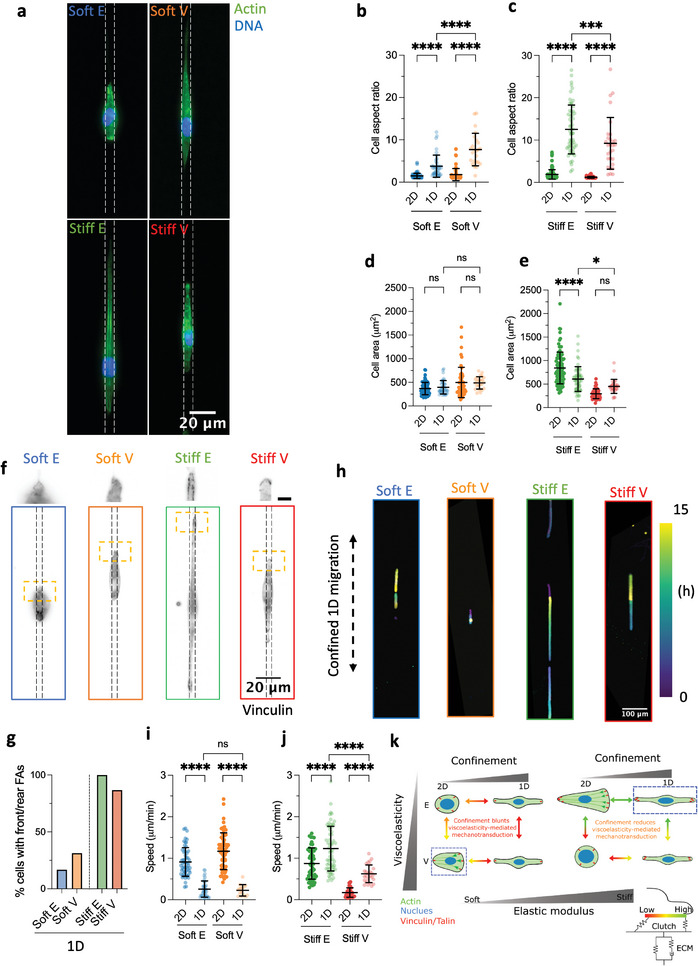
Spatial confinement blunts viscoelasticity‐mediated effects on soft matrices and reduces them on stiff matrices. a) Representative Actin/DNA images of confined MCF‐10A cells on 5 µm FN lines on elastic and viscoelastic PAAm hydrogels. FN lines are schematically represented as dashed lines for clarity. b) Quantification of MCF‐10A cell aspect ratio on 2D and 1D soft elastic (Soft E) and viscoelastic (Soft V) matrices (*n* = 74 cells for Soft E 2D, *n* = 40 cells for Soft E 1D, *n* = 50 cells for Soft V 2D, *n* = 24 cells for Soft V 1D) from at least two independent experiments. ^****^ *p* < 0.0001, two‐way ANOVA with Tukey's multiple comparisons test. c) Quantification of MCF‐10A cell aspect ratio on 2D and 1D stiff elastic (Stiff E) and viscoelastic (Stiff V) matrices (*n* = 100 cells for Stiff E 2D, *n* = 59 cells for Stiff E 1D, *n* = 48 cells for Stiff V 2D, *n* = 29 cells for Stiff V 1D) from at least two independent experiments. ^****^ *p* < 0.0001, ^***^ *p* = 0.0006, two‐way ANOVA with Tukey's multiple comparisons test. d) Quantification of MCF‐10A cell spreading area on 2D and 1D soft elastic (Soft E) and viscoelastic (Soft V) matrices (*n* = 74 cells for Soft E 2D, *n* = 40 cells for Soft E 1D, *n* = 50 cells for Soft V 2D, *n* = 24 cells for Soft V 1D) from at least two independent experiments. ns *p* > 0.05, two‐way ANOVA with Tukey's multiple comparisons test. e) Quantification of MCF‐10A cell spreading area on 2D and 1D stiff elastic (Stiff E) and viscoelastic (Stiff V) matrices (*n* = 100 cells for Stiff E 2D, *n* = 59 cells for Stiff E 1D, *n* = 48 cells for Stiff V 2D, *n* = 29 cells for Stiff V 1D) from at least two independent experiments. ^****^ *p* < 0.0001, ^*^ *p* = 0.0473, ns *p* = 0.0588, two‐way ANOVA with Tukey's multiple comparisons test. f) Representative FAs (Vinculin) images of confined MCF‐10A cells on 5 µm FN lines on elastic and viscoelastic PAAm hydrogels. FN lines are schematically represented as dashed lines for clarity. Scale bar in the inset is 5 µm. g) Percentage of cells forming front and rear vinculin adhesions pooled from two independent experiments (*n* = 13 cells for Soft E, *n* = 17 cells for Soft V, *n* = 31 cells for Stiff E, *n* = 16 cells for Stiff V). h) Representative temporal color‐coded time lapses of MCF‐10A migrating on 5 µm FN lines. The direction of confined migration is shown by a dashed line. i) Quantification of MCF‐10A cell migration speed on 2D and 1D soft elastic (Soft E) and viscoelastic (Soft V) matrices (*n* = 56 cells for Soft E 2D, *n* = 38 cells for Soft E 1D, *n* = 61 cells for Soft V 2D, *n* = 14 cells for Soft V 1D) from at least two independent experiments. ^****^ *p* < 0.0001, ns *p* = 0.9911, two‐way ANOVA with Tukey's multiple comparisons test. j) Quantification of MCF‐10A cell migration speed on 2D and 1D stiff elastic (Stiff E) and viscoelastic (Stiff V) matrices (*n* = 55 cells for Stiff E 2D, *n* = 61 cells for Stiff E 1D, *n* = 35 cells for Stiff V 2D, *n* = 26 cells for Stiff V 1D) from at least two independent experiments. ^****^ *p* < 0.0001, two‐way ANOVA with Tukey's multiple comparisons test. k) Schematic representation of how confinement modulates viscoelasticity sensing compared to 2D matrices. Colorful arrows show how clutch sensitivity (as indicated by the measured outputs) to ECM viscoelastic properties (here schematically represented by a standard linear solid lumped model) changes in each condition. Overall, confinement blunts ECM viscoelasticity mechanotransduction effects on soft ECMs and reduces them on stiff ECMs. Dashed blue boxes show conditions where optical migration occurs (Soft V 2D and Stiff E 1D). For all plots in this figure the central bar represents the mean, whereas the error bars represent the SD.

To address this gap, we micropatterned 5 µm FN lines on all substrates (Figure 1h), corresponding to the upper threshold above which cells have been shown to lose their uniaxial 1D spindle‐like morphology.^[^ ^23^ ^]^ We first studied whether 1D confinement and viscoelasticity could affect the morphology of breast epithelial cells in terms of spreading area and aspect ratio. Regardless of matrix viscoelastic properties, our findings show that cells on 5 µm lines adopted a 1D spindle‐like morphology with a high aspect ratio (**Figure** **4**a–c) compared to 2D conditions (Figure 2a). However, 1D confinement affected cell spreading area differentially depending on ECM viscoelasticity. Specifically, on soft substrates, cell spreading area was not affected by confinement (Figure 4d). Conversely, cell spreading area was significantly reduced on 1D Stiff E matrices compared to their 2D counterparts, consistent with results reported on stiff elastic substrates^[^ ^23^, ^78^ ^]^ (Figure 4e), indicating that cells are restricted when confined to 1D lines on Stiff E matrices. Similarly to soft substrates, cell spreading area was not affected on Stiff V matrices as compared to their 2D counterpart. However, cells became elongated and spread along the pattern compared to their circular unpolarized morphology on 2D matrices (Figure 4e).

When comparing cell aspect ratio under confined 1D conditions, we observed that cells exhibited greater elongation on Soft V hydrogels compared to their elastic counterparts (i.e., Soft E) (Figure 4b). Conversely, cells displayed increased elongation on Stiff E conditions compared to their viscoelastic (i.e., Stiff V) counterparts. These findings suggest that breast epithelial cells adapt their morphology in response to the viscoelastic properties of the matrix even under confinement. Importantly, when assessing cell spreading area within 1D confined conditions, we found no significant differences between Soft E and Soft V matrices (Figure 4d), suggesting that spatial confinement blunts viscoelasticity‐mediated effects observed on soft 2D matrices. Additionally, although cells on 1D Stiff E matrices remained larger than those on 1D Stiff V matrices, this was to a lesser extent than observed in 2D conditions (Figure 4e). This indicates that confinement reduces the influence of underlying matrix viscoelasticity on cell spreading area when the stiffness of the matrix is elevated. Overall, 1D confinement compels cells to adopt a similar spreading area, characterized by an elongated spindle‐like morphology.

We further investigated how spatial confinement affects FAs on viscoelastic ECMs, focussing on the spatial localization of FAs. The formation and disengagement of front‐rear FAs have been associated with the ability of cells to break symmetry and migrate when confined on 1D lines on stiff elastic PAAm matrices via a stick‐slip mechanism.^[^ ^41^ ^]^ Regardless of matrix viscoelasticity, cells on soft matrices rarely formed front‐rear FAs, whereas most cells on stiff matrices were able to form front‐rear adhesions (Figure 4f,g). To complement FA data obtained via immunofluorescence, we again transfected MCF‐10A cells with talin‐GFP to visualize FA dynamics on micropatterned viscoelastic substrates, similar to 2D substrates. As anticipated, cells on soft E/V substrates lacked prominent adhesions, failed to break symmetry and remained stationery over the 30‐min imaging period (Movies S17–18, Supporting Information). In contrast, cells on Stiff E/V substrates exhibited dynamic FA disassembly on one side, enabling migration in the opposite direction (Movies S19–S20, Supporting Information).

Interestingly, these morphological and FA changes did not strongly change YAP localization. YAP which either remained constant or slightly decreased compared to its localization on 2D unconfined ECMs (Figure S11, Supporting Information).

We then investigated how the observed morphological changes and adhesion phenotype would affect 1D cell migration on viscoelastic ECMs. Migration along 1D lines is an accepted reductionist model to mimic in vivo migration conditions involving confined spaces, such as within dense tissues, along blood vessels, ECM fibres or muscle fibres.^[^ ^24^ ^]^ However, 1D lines are usually produced on stiff glass coverslips^[^ ^78^ ^]^ or at most on elastic PAAm hydrogels with stiffness in the range of tens of kPa,^[^ ^41^ ^]^ leaving unexplored how matrix‐mimetic viscoelastic properties affect 1D confined cell migration on soft substrates. We therefore followed cell migration using time lapse microscopy for 15 h and observed that migration was significantly increased for cells on stiff 1D ECMs compared to their 2D counterparts, as indicated by a significant increase in speed (Figure 4h–j and Movies S21–S22, Supporting Information). Conversely, cell migration speed was significantly decreased on 1D Soft E and Soft V matrices compared to 2D unconfined conditions (Figure 4h–j and Movies S23–S24, Supporting Information). Consistent with our 2D findings, faster cells demonstrated greater directional persistence, as quantified by the number of direction changes per hour (Figure S12, Supporting Information). Cells on Soft E/V matrices oscillated around their centre of mass, whereas those on Stiff E/V matrices broke their symmetry and migrated more persistently in a specific direction. Previous studies have reported that confinement can either enhance^[^ ^23^ ^]^ or hinder^[^ ^26^, ^78^ ^]^ cell migration on stiff substrates, depending on the cell type. Furthermore, the relationship between cell speed and confinement has been shown to depend on ECM stiffness.^[^ ^25^, ^30^ ^]^ Our findings align with these results, where a decrease in migration was observed in soft and narrow collagen microchannels compared to wider channels of similar mechanical properties,^[^ ^30^ ^]^ and conversely, an increase in migration speed was observed in stiff and narrow collagen microchannels compared to wider channels of similar mechanical properties.^[^ ^30^ ^]^

When comparing cell migration within 1D conditions, our results demonstrate that 1D spatial confinement elicits opposite effects on cell migration depending on the stiffness of the matrices, consistent with previous findings.^[^ ^30^ ^]^ However, we additionally demonstrate that spatial confinement blunts viscoelasticity‐mediated migration effects in soft 1D ECMs (Figure 4i), consistent with how confinement abrogates 2D viscoelasticity‐driven differences in cell spreading area when the stiffness of the matrix is low. At high matrix stiffness, we observed a decreased difference in migration speed between cells on Stiff E and Stiff V matrices compared to 2D conditions (Figure 4j), again showing that spatial confinement modulates how matrix viscoelasticity is transduced into dynamic migration processes. These findings are schematically summarized in Figure 4k.

The inability of breast epithelial cells on soft 1D matrices to form front rear FAs implies a deficiency in breaking their own symmetry and polarize, thus impairing their migration. This process is insensitive to matrix viscoelasticity. In contrast, cells on stiff substrates can stochastically break their symmetry by disassembling front‐rear FAs, initiating migration depending on the viscoelastic properties of the underlying matrix. These findings are consistent with a 1D motor clutch‐based model, emphasizing the importance of asymmetry in the number of bound clutches between both cell extremities.^[^ ^41^ ^]^ At the cell end with fewer bound molecular clutches, a higher traction force per clutch is sustained, resulting in catastrophic rupture of the cell‐substrate adhesions on this side and a subsequent translocation of the cell toward the opposite side.

We anticipate that integrating ECM viscoelastic properties across various stiffness regimes into models describing 1D cell migration will offer insights into how migration mechanisms transition from 2D unconfined to 1D confined conditions, and eventually to 3D confinement.

## 3 Conclusions and Outlook

This study presents a robust and adaptable method for manipulating elasticity, viscoelasticity, and micropatterning (spatial confinement) within PAAm hydrogels. While previous works on viscoelastic matrices^[^ ^13^, ^14^, ^21^ ^]^ have predominantly focused on elasticity ranges above 2–3 kPa, we designed viscoelastic hydrogels with remarkably low elasticities (0.3 ≤ *E* ≤ 3 kPa) to accurately mimic specific aspects of breast cancer progression, such as the transition from a soft viscoelastic matrix to a stiff elastic matrix.^[^ ^35^, ^38^ ^]^

Mechanistically, our findings revealed that enhanced viscoelasticity exerts contrasting effects on cell spreading, FAs, YAP nuclear import and cell migration depending on substrate stiffness. Notably, FA formation and YAP nuclear import remained highly responsive to ECM viscoelastic properties, displaying a linear correlation with cell spreading area. These findings underscore the pivotal role of cell contractility in mediating YAP nuclear translocation in response to matrix viscoelastic properties, complementing the well‐established essential involvement of YAP in various aspects of breast cancer development.^[^ ^79^ ^]^ While previous studies have emphasized the role of matrix stiffness in the regulation of cell mechanobiology during breast tumor progression,^[^ ^38^, ^39^, ^56^ ^]^ our study highlights the pivotal role of substrate viscoelasticity in regulating cell mechanotransduction and migration in this context. Specifically, we demonstrate that viscoelasticity enhances cell mechanotransduction and migration on soft substrates, while impeding these processes on stiff substrates, via regulation of actin and FA dynamics.

Although these results were obtained in two dimensions, the combination of viscoelastic hydrogels with protein micropatterning provides a facile and reproducible platform to further investigate the interplay between spatial confinement and viscoelasticity and to capture certain aspects of 3D cell migration.^[^ ^23^ ^]^ Our findings indicate that spatial confinement reduces viscoelasticity‐mediated differences in cell spreading area and cell migration, while propelling cells to adopt an elongated spindle‐like morphology with reduced adhesions. Altogether, these findings suggest a complex interplay between viscoelastic properties and dimensionality of the surrounding matrix in the regulation of cell migration and mechanotransduction. Our results derived from 2D experiments with very low elasticities are consistent with a motor clutch‐based model substrate wherein viscoelasticity regulates the lifetimes and the number of engaged clutches.^[^ ^21^ ^]^ However, they also indicate that heightened levels of spatial confinement obscure the viscoelastic properties of the substrate, particularly at extremely low stiffness values, by limiting the distribution of cell‐substrate interactions, which constitute a critical aspect of the molecular clutch model.^[^ ^59^ ^]^

These findings highlight the significance of incorporating stress relaxation as a critical parameter when studying complex cellular behaviors and mechanotransduction signals within human tissues. This approach provides a platform to further decouple the elastic modulus from matrix viscoelastic properties and the level of imposed spatial confinement, without confounding effects introduced either by ligand density variations or matrix viscoplasticity.

We anticipate that that the developed system will be useful in a variety of applications in the field of mechanobiology where matrix‐mimetic viscoelastic properties can be combined with different confining geometries to study dynamic single and multi‐cellular mechanobiological processes. A limitation of our current work is how cell–cell contacts, an essential feature of epithelial tissues,^[^ ^80^ ^]^ would affect how matrix viscoelastic properties are sensed and transduced.^[^ ^81^, ^82^ ^]^ The use of larger micropatterns for the study of collective epithelial behavior on the developed viscoelastic gels is an exciting opportunity of further research to explore the role of ECM viscoelasticity in more physiologically relevant systems.

Similarly, whereas the thin FN micropatterns employed in this work replicate the 3D fibrillar environment of the native ECM to a certain extent,^[^ ^23^ ^]^ 3D cell‐culture systems are starting to unravel additional mechanisms of mechanotransduction mediated by 3D confinement.^[^ ^83^ ^]^ While not addressed in the current study, our system opens the possibility to investigate how viscoelasticity mechanotransduction changes from 2D to 3D in a well‐controlled experimental platform, for example through the 3D microstructurization of the developed hydrogels.^[^ ^84^ ^]^

## 4 Experimental Section

### Coverslips Silanization

The silanization procedure for coverslips followed previously established protocols.^[^ ^43^, ^85^ ^]^ Briefly, 22 mm coverslips (VWR) were incubated with 0.1 m NaOH for 5 min, followed by three rinses in Milli‐Q water, the last time for 5 min. Subsequently, coverslips were dried using a gentle nitrogen flow. Next, 15 µL of 3‐(Acryloyloxy)propyltrimethoxysilane (Alfa Aesar) per coverslip was pipetted onto a glass plate, and each coverslip was flipped to place the activated side on the drop for 1 h. Afterwards, coverslips were then rinsed three times in Milli‐Q water, gently dried with nitrogen, and stored at 4 °C until further use.

### Fabrication of Polyacrylamide Hydrogels

All reagents for PAAm hydrogels were purchased from Merck. PAAm hydrogels with adjustable viscoelastic properties were fabricated by mixing varying amounts of 40% AAm, 2% Bis and Milli‐Q water in a 1 mL Eppendorf tube. The solutions were gently vortexed to ensure thorough mixing without introducing oxygen. To introduce reactive aldehyde groups for protein binding, a solution of oxidized N‐hydroxyethyl acrylamide (OHEA) was prepared as previously outlined^[^ ^52^ ^]^ and an equal volume of 10 µL added to each prepolymer solution. Each Eppendorf was again gently vortexed to ensure homogeneous mixing of the OHEA within the prepolymer solutions. To initiate polymerization, the same volumes of 100% tetramethylethylenediamine (TEMED) (2.5 µL), 10% ammonium persulfate (APS) in milli‐Q water (7.5 µL) were added to each Eppendorf. The solutions were gently vortexed one last time. For the formation of thin hydrogel films immobilized on glass coverslips, 30 µL of each prepolymer solution was pipetted on a hydrophobic flexible PCTFE sheet (Agar Scientific) treated with plastic RainX (Figure S1, Supporting Information) and covered with a 22 mm acrylsilanized glass coverslip. Gelation was allowed to occur for 30 min at room temperature, after which the PCTFE‐coverslip sandwich was submerged in cold (4 °C) milli‐Q water for 1 h before peeling the PCTFE sheet off. Hydrogels were then rinsed three times with milli‐Q water to remove any unreacted groups and left to swell at 4 °C overnight in milli‐Q water before protein functionalization. Hydrogel compositions are summarized in Table S1 (Supporting Information).

### Homogeneous Protein Functionalization

Following swelling, hydrogels underwent a thorough rinsing three times with Milli‐Q water. Excess water was gently removed from the hydrogel surface by blotting the side of each coverslip against tissue paper, ensuring that the hydrogel surfaces were not completely dry. Subsequently, 100 µL of 70 µg mL^−1^ (high concentration) or 10 µg mL^−1^ (low concentration) solution of human FN (Yo Proteins) in Milli‐Q water was pipetted onto the hydrogel surfaces and allowed to evaporate under airflow. This process facilitated the covalent binding of the primary amines of the FN with the aldehyde groups present in the PAAm hydrogels.^[^ ^51^, ^52^ ^]^ The samples were then rehydrated in Milli‐Q water for 5 min and rinsed three times with Milli‐Q water to eliminate unbound protein.

### Microstamp Fabrication and Protein Micropatterning

Microstamps featuring 5 µm lines were fabricated using a silicon master obtained through deep reactive‐ion etching from a chromium photomask (Toppan Photomask). The silicon master underwent passivation with a fluorosilane (tridecafluoro‐1,1,2,2‐tetrahydrooctyl‐1‐trichlorosilane; Gelest) for 30 min in a desiccator. Subsequently, polydimethylsiloxane (PDMS) (Sylgard 184 Silicone Elastomer Kit; Dow Corning) mixed at 10:1 w:w ratio (base:cross‐linker) was poured onto the master to achieve a height of approximately 1 cm and degassed to eliminate any bubbles. The PDMS was cured for a minimum of 4 h at 60 °C. Following this, the PDMS layer was carefully detached from the master, and stamps cut into approximately 1 cm^3^ cubes. PAAm hydrogels were microprinted based on a previously published protocol.^[^ ^43^, ^85^ ^]^ Briefly, stamps were sonicated in a solution of 5% decon 90 (decon) in milli‐Q water for 15 min, thoroughly rinsed under water, and sonicated again in a solution of 70% isopropanol in Milli‐Q water for 15 min. The stamps were then gently dried under nitrogen flow. PDMS stamps were activated using an ultraviolet/O_3_ oven (UV–Ozone photoreactor PR‐100, UVP Products) for 8 min and incubated with a solution of 70 µg mL^−1^ FN in Milli‐Q water for 1 h at room temperature. Subsequently, PDMS stamps were gently dried under nitrogen and placed in contact with dried hydrogel surfaces for 1 h at room temperature. To facilitate stamp detachment from hydrogels, hydrogels/stamps composites were submerged in Milli‐Q water for 10 min, and stamps carefully removed. To visualize the integrity of patterns during protocol optimization using fluorescence microscopy, 35 µg mL^−1^ of Alexa Fluor 488 or 647 fibrinogen (ThermoFisher) were added to the FN solution.

### Determination of the Young's Modulus

Nanoindentation measurements were conducted using a commercially available fibre‐optic based nanoindentation device (Chiaro, Optics 11 Life)^[^ ^8^, ^86^ ^]^ mounted on top of an inverted optical microscope (Axiovert 200 M, Zeiss), following a standardized protocol.^[^ ^49^ ^]^ The hydrogel *E* was determined by moving the probe at a constant speed of 2 µm s^−1^ over a vertical range of 10 µm (displacement control). For soft hydrogels, a cantilever with stiffness (*k*) of 0.028 N m^−1^ equipped with a spherical bead of 26 µm in radius (*R*) was used. In the case of stiff hydrogels, a cantilever with a *k* of 0.52 N m^−1^ and *R* of 27.5 µm was employed. The forward segment of the collected force–displacement (*F–z*) curves was analyzed using an open‐source software.^[^ ^49^ ^]^ To convert *F*–*z* curves into force‐indentation (*F*–δ) curves, the contact point was found using a goodness‐of‐fit algorithm.^[^ ^87^ ^]^ Subsequently, the Hertz model (Equation 1) was fitted up to an indentation of 10% of the probe radius (i.e., δ = 0.1*R*) to obtain *E*, assuming Poisson's ratio (ν) to be 0.5:

(1) $$F=\frac{4}{3}\frac{E}{\left(1-v^{2}\right)}\delta^{\frac{3}{2}}R^{\frac{1}{2}}$$

At least three hydrogels per condition were tested, with a minimum of 50 indentations per hydrogel. Each indentation was spaced at least twice the contact radius ($a=\sqrt{\delta R}$) from the previous one to ensure testing a different point. All measurements were conducted at room temperature in Milli‐Q water on thin hydrogel films immobilized on silanized coverslips.

### Determination of the Stress Relaxation

Stress relaxation measurements were conducted using a commercially available fibre‐optic based nanoindentation device (Chiaro, Optics 11 Life)^[^ ^8^, ^86^ ^]^ mounted on top of an inverted optical microscope (Axiovert 200 M, Zeiss), adapting a previously described approach.^[^ ^18^ ^]^ The instrument operated in closed‐loop mode (Indentation mode), maintaining a constant indentation depth, δ, over time to characterize the local stress relaxation response of the material. A cantilever with a *k* of 0.52 N m^−1^ and *R* of 27.5 µm was employed for all hydrogels. Hydrogels underwent indention at a strain rate of 30 µm s^−1^ until an indentation depth of 3 µm was reached (about 10% of the probe radius, *R*), maintained for 60 s. This corresponds to an applied strain (ε) of ≈7% given^[^ ^88^ ^]^:

(2) $$\varepsilon =0.2\frac{\sqrt{R\delta }}{R}$$

which fulfils the small strain approximation.^[^ ^88^ ^]^ Concurrently, the load signal was recorded. The acquired data were analyzed using a previously described open‐source Jupyter notebook available on GitHub.^[^ ^18^ ^]^ At least two hydrogels per conditions were tested, with over 100 points per hydrogel, spaced at least twice the contact radius ($a=\sqrt{\delta R}$) from the previous one to ensure different points were tested. All measurements were performed at room temperature in Milli‐Q water on thin hydrogel films immobilized on silanized coverslips.

### Bulk Rheological Characterization

Bulk rheology measurements were conducted using a Modular Compact Rheometer (MCR 302e, Anton Paar) equipped with a parallel plate geometry. A 15 mm diameter upper plate was employed for all tests. In brief, bulk gels were prepared by dispensing 400 µL of the pre‐polymer solution on a PCTFE sheet (Agar Scientific), which was then covered with a hydrophobic glass coverslip (treated with RainX solution, RainX) of 18 mm in diameter. This process resulted in hydrogels of approximately 18 mm in diameter with a thickness of ≈1 mm. The hydrogels were allowed to polymerize for 1 h at room temperature. Subsequently, the coverslip‐hydrogel‐PCTFE sandwich was immersed in cold Milli‐Q water for 1 h, and hydrogels were gently detached. After washing the hydrogels three times in Milli‐Q water, they were stored at 4 °C overnight in Milli‐Q water to facilitate swelling. Prior to measurements, hydrogels were punched to 15 mm to match the rheometer's upper plate dimensions. The upper plate of the rheometer was brought into contact with the sample until a normal force of ≈ 0.1 N was reached to ensure good contact without introducing compressional stiffening effects.^[^ ^89^ ^]^ Subsequently, an amplitude sweep between 0.01% and 1% strain (angular frequency of 10 rad s^−1^) was performed to identify a suitable strain within the linear viscoelastic regime for subsequent frequency sweeps. A frequency sweep was then conducted at 1% strain between 0.0186 and 8.6100 Hz (0.1 to 54 rad s^−1^). All measurements were performed at room temperature in Milli‐Q water, and the hydration of the samples was maintained using a solvent trap. Three independent samples per condition were tested.

### Cell Culture

MCF‐10A cells were cultured at high confluency in DMEM/F‐12 (Thermo Fisher) supplemented with 5% Horse Serum (Thermo Fisher), 20 ng mL^−1^ epidermal growth factor (EGF) (Peprotech), 0.5 µg mL^−1^ Hydrocortisone (Merck), 100 ng mL^−1^ Cholera Toxin (Enzo Life Sciences), 10 µg mL^−1^ insulin from bovine pancreas (Merck) and 0.1% Penicillin/Streptomycin (Pen/Strep) (Merck). Cells were passaged every 3–4 days when reaching full confluence and plated at a 1:4 dilution (≈2 million cells/T75 flask). For cell passaging, the culture media was aspirated, and cells were washed with 10 mL of 1× PBS. After aspirating the PBS, cells were incubated with 2 mL of 0.25% trypsin (Merck) at 37°C for 10 min. Trypsinization was halted by adding 4 mL of media, and cells were spun at 1.3 rpm for 5 min in a 15 mL falcon tube to obtain a pellet. The old media was aspirated, and cells were resuspended in 1 mL of fresh media before being plated. Cells were used up to a maximum of 15 passages.

### Cell Plating on 2D and 1D Substrates

Prior to protein functionalization and cell seeding, both PAAm hydrogels and glass coverslips (VWR, 170 µm thick) underwent UV sterilization for 30 min. Cells were trypsinized following the cell culture protocol and then plated at 20 000 cells per gel (approximately 7000 cells mL^−1^ or roughly 5000 cells cm^−2^, considering a 22 mm hydrogel surface). After plating, cells were allowed to adhere for 24 h at 37 °C with 5% CO_2_ before initiating any experiment. To mitigate epithelial to mesenchymal transition (EMT),^[^ ^90^ ^]^ cells were maintained in monolayer cultures in tissue flasks before plating on the hydrogels. Although we performed our experiments at short time points (24 h), it has been shown that sparse culture conditions can influence the expression of EMT marker genes at similar time points.^[^ ^91^ ^]^ This was not formally tested in our system given that EMT is often partially and transiently activated, and a full mesenchymal state cannot be defined.^[^ ^90^ ^]^

### Cell Migration Experiments

MCF10‐A cells were seeded following the procedure outlined above. To arrest cell division before commencing time‐lapse experiments, cells were treated with 5 µg mL^−1^ Mitomycin C (Merck) for 1 h. Subsequently, Mitomycin C was removed through a single wash with fresh media, coverslips were transferred in an Attofluor chamber (ThermoFisher), and fresh media was added before initiating imaging. Time‐lapse experiments were conducted on either a Nikon C1 or a Nikon Ti2 A1R HD25 inverted microscope (Nikon, Japan) using either a 20×/0.45 or 20×/0.75 DIC objective, respectively. Images were taken at 3‐min intervals over a 15‐h period. An incubation chamber was employed to maintain CO_2_ levels at 5% and temperature at 37°C during imaging. Multiple positions (*N* > 5) were imaged in all cases using a motorized stage. Each condition underwent at least two independent experiments.

### Immunocytochemistry

Staining of samples on both hydrogels and glass coverslips was carried out following the same protocol. Samples were washed three times with PBS and fixed with 4% Paraformaldehyde in PBS for 12 min at room temperature either after cell migration experiments or at least after 24 h of plating cells. Subsequently, samples were then washed three times with PBS, with the last wash lasting for 5 min, and permeabilized using 0.05% Triton X‐100 in PBS for 10 min at room temperature. Following another round of three washes with PBS, samples were blocked with a solution of 5 v/v % fetal bovine serum (FBS, Gibco) and 1 w/v % Bovine Serum Albumin (BSA, Merck) in PBS for 30 min at room temperature. After an additional three washes with PBS, a solution of 1 w/v % BSA in PBS containing the primary antibody, Alexa Fluor 488 Phalloidin 1:200 (Thermofisher) and DAPI 1:200 (Thermofisher) was added to the samples and incubated for 45 min at 37°C. We utilized either the monoclonal anti‐vinculin antibody produced in mouse (Merck, Ref: V9131) at 1:200 or the YAP monoclonal antibody (M01 clone 2F12, Abnova, Ref: H00010413‐M01) at 1:100 as primary antibodies. Subsequently, samples were washed three time with PBS, and incubated with goat anti‐mouse Alexa Fluor 555 secondary antibody (Thermofisher) (dilution 1:200) or Cy3‐conjugated Rabbit Anti Mouse (Jackson ImmunoResearch) (dilution 1:250) in a solution of 1 w/v % BSA in PBS for 45 min at 37°C. Following another round of three washes with PBS, samples were mounted using ProLong Diamond Antifade Mountant (Thermofisher) and stored at 4°C until imaging.

### Image Acquisition for Fixed Samples

Images of the actin cytoskeleton on 2D homogeneous substrates were captured using either a Nikon C1 inverted fluorescence microscope with a ×20/0.45NA Plan Fluor objective and ×60/1.4 Plan Apo oil immersion objective; or a Zeiss LSM 980 confocal microscope using a ×20/0.8 Plan Apo or ×63/1.4 Plan Apo oil immersion objective. Vinculin images for homogeneous 2D substrates were acquired using a Nikon C1 inverted fluorescence microscope with a 60×/1.4 Plan Apo oil immersion objective. YAP images for homogeneous 2D or micropatterned 1D substrates were acquired using a Zeiss LSM 980 confocal microscope using a ×20/0.8 Plan Apo objective or ×63/1.4 Plan Apo oil immersion objective. Actin cytoskeleton images on 1D micropatterned substrates were captured using a Nikon C1 orTi2 A1R HD25 inverted microscope (Nikon, Japan) with a ×60/1.4 Plan Apo objective or ×100/1.35 Plan Apo silicone immersion objective. Vinculin images on 1D micropatterned substrates were obtained using a Nikon Ti2 A1R HD25 inverted microscope equipped with a 100×/1.35 Plan Apo silicone immersion objective. When using confocal microscopy for quantification of YAP translocation, the pinhole was opened to 1.5 Airy Units so that the collected signal originated from the entire cell volume.

### Actin Flow Experiments

MCF‐10A cells were plated on PAAm hydrogels, following the previously described protocol for cell plating on 2D PAAm hydrogels. Prior to imaging, cells were incubated with SPY555‐FastAct (Spirochrome) for 2 h at 37°C following the manufacturer's protocol (dilution 1:1000 in culture medium). The hydrogels were then transferred to an Attofluor chamber (Thermofisher). Live actin imaging was performed on a Zeiss LSM 980 confocal microscope using a Plan Apo 40×/1.3 oil immersion objective. An incubation chamber was used to maintain CO_2_ levels at 5% and temperature at 37°C throughout the imaging session. Images were acquired at a rate of one per second for a total duration of 2 min.

### Live Talin Experiments

MCF‐10A cells (500 × 10^3^) were seeded in a T25 flask containing 3 mL full culture medium with low serum (1% instead of 5%) and 150 µL of CellLight Talin‐GFP, BacMam 2.0 (Thermofisher). Cells were incubated overnight at 37°C in a humified atmosphere with with 5% CO_2_. Following incubation, cells were harvested and plated on hydrogels as previously described. Time lapse imaging was performed using a Ti2 A1R HD25 inverted microscope (Nikon, Japan) equipped a 100×/1.35 Plan Apo silicone immersion objective. An incubation chamber was used to maintain stable conditions of 5% CO_2_ and 37°C throughout the imaging session. Images were captured every 15 s for homogeneous substrates and every 50 s for micropatterned substrates, with a total acquisition time of 30 min in both conditions.

### Cell Projected Area and Cell Morphology

Cell projected area, circularity, and aspect ratio were quantified in Fiji^[^ ^92^ ^]^ by applying a Gaussian blur filter (sigma = 2), followed by a default threshold to segment individual cells and quantify parameters of interest. The threshold was manually adjusted to capture the entire cell cytoskeleton.

### Focal Adhesions Analysis

FAs were analyzed in Fiji^[^ ^92^ ^]^ by following a previously published protocol.^[^ ^93^ ^]^ Briefly, images were cropped to include only one cell per image. Subsequently, background subtraction was performed using a sliding paraboloid and rolling ball radius of 50. Local contrast was then enhanced by applying CLAHE using block size = 19, histogram bins = 256, and maximum slope = 6. The image underwent an exponential transformation to minimize background. Automatic adjustments were made to image brightness and contrast, followed by the application of a Log3D filter with sigmax = sigmay = 3 applied. The LUT of the image was inverted, and automatic threshold was applied to convert the image to a binary. A watershed algorithm was then used to eliminate incorrectly clustered adhesions. Finally, the “Analyze Particles” command was executed (minimum 20 pixels to maximum infinity) to quantify FAs area and count per cell. Alternatively, for 1D micropatterned hydrogels, images were inspected to count the number of cells having front/rear vinculin patches.

### Nuclear to Cytoplasmic YAP Ratio

Nuc/Cyto YAP ratio was quantified using Fiji.^[^ ^92^ ^]^ Initially, nuclear area (*A*_nuc_) was calculated by applying a gaussian blur (sigma = 2) and segmenting nuclei (DAPI channel) via the default threshold. Subsequently, cell area (*A*_cell_) was calculated by applying a gaussian blur (sigma = 2) and segmenting the actin cytoskeleton (Phalloidin channel) via the Default threshold. Nuclear and cellular outlines were saved in the Fiji ROI manager and utilized to quantify integrated density of the cell (YAP_cell_) and the nucleus (YAP_nuc_) in the YAP channel. Therefore, Nuc/Cyto YAP ratio was calculated as follows:

(3) $$\mathrm{YA}P_{\frac{\mathrm{nuc}}{\mathrm{cyto}}}=\frac{\left(\frac{\mathrm{YA}P_{\mathrm{nuc}}}{A_{\mathrm{nuc}}}\right)}{\left(\frac{\mathrm{YA}P_{\mathrm{cyto}}}{A_{\mathrm{cyto}}}\right)}$$

where

(4) $$\mathrm{YA}P_{\mathrm{cyto}}=\mathrm{YA}P_{\mathrm{cell}}-\mathrm{YA}P_{\mathrm{nuc}}$$

and

(5) $$A_{\mathrm{cyto}}=A_{\mathrm{cell}}-A_{\mathrm{nuc}}$$

Although this is an approximation as cells are 3D objects in 3D space, the fluorescence signal is integrated over the cell volume (see Image acquisition for fixed samples) and thus represents an accurate approximation of its spatial localization when taking the ratio between Nuc and Cyto regions.^[^ ^94^ ^]^ This is a standard approach to study the translocation of YAP in studies involving 2D substrates.^[^ ^65^, ^68^ ^]^

### Cell Tracking and Migration Analysis

DIC time‐lapses were imported in Fiji,^[^ ^92^ ^]^ converted to 8‐bit format, and analyzed using CellTracker.^[^ ^95^ ^]^ In brief, manual tracking was performed by clicking on the cell's centroid every two frames, and linear interpolation was applied to compute their position over time. Only cells that migrated a distance greater than three cell bodies were considered migratory and therefore tracked. Cell trajectories were aligned to a common origin and are shown for a tracking time of 5 h. Cell speed was computed as the mean instantaneous speed considering the entire tracking period. For a tracking time of 5 h, MSD, directionality ratio, and autocorrelation were computed using a previously published protocol^[^ ^71^ ^]^. Average MSD curves were fitted according to:

(6) $$\mathrm{MSD}\left(t\right)=4\times D\times \tau^{\alpha }$$

In Equation (6), *α* represents the anomalous diffusion exponent describing the type of diffusion of the system (*α* = 1 for a diffusive system, *α* > 1 for a super diffusive system and *α* < 1 for a sub diffusive system), *τ* is the lag time and *D* is the diffusion coefficient.^[^ ^21^, ^96^ ^]^

### Retrograde Actin Flow

To determine the retrograde actin flow at the cell leading edge (lamellipodium), time‐lapses were initially converted to 8‐bit format, underwent background subtraction, contrast enhanced, and were subjected to a Gaussian blur filter (sigma = 1.5). Subsequently, kymographs were generated using the Multi Kymograph plugin in Fiji with a line width of 1. Actin retrograde flow speed was then computed using the bounding rectangle parameters as follows:

(7) $$v=\frac{\frac{\mathrm{width}}{c_{1}}}{\frac{\mathrm{height}}{c_{2}}}$$

where width is the width of the bounding rectangle in pixels, *c*_1_ is the spatial conversion factor (px µm^−1^), height is the height of the bounding rectangle in pixels, and *c_2_* is the temporal conversion factor (px s^−1^). This calculation yields the actin retrograde flow speed (*v*) in units of µm s^−1^, which is subsequently converted to nm s^−1^.

### Statistical Analysis

All statistical analyses were performed in Prism v10 (GraphPad). Two‐tailed unpaired student t tests were employed when comparing two conditions. For comparisons involving more than two conditions with variations in two variables, two‐way ANOVAS with Bonferroni's or Tukey's multiple comparisons tests were performed. For comparisons involving more than two conditions with variations in three variables, three‐way ANOVAS were performed with Tukey's multiple comparisons test. Specific tests conducted for each analysis are detailed in the respective figure captions. Comparisons were considered significant when the *p* value was at least smaller than 0.05.

## Conflict of Interest

The authors declare no conflict of interest.

## Author Contributions

SG, GC and MS‐S conceived the project. SG, MS‐S, MC and MV (University of Glasgow) supervised the project. GC performed all experiments and analyzed all data. MAGO performed nanoindentation experiments together with GC. MV (University of Mons) contributed to microprinting and imaging experiments. GC wrote the first original draft of the article, which was reviewed and edited by SG and MS‐S. The article was read and corrected by all authors, who contributed to the interpretation of results. Funding was acquired by MV (University of Glasgow), MS‐S and SG.

## Supporting information



Supporting Information

Supplemental Movie 1

Supplemental Movie 2

Supplemental Movie 3

Supplemental Movie 4

Supplemental Movie 5

Supplemental Movie 6

Supplemental Movie 7

Supplemental Movie 8

Supplemental Movie 9

Supplemental Movie 10

Supplemental Movie 11

Supplemental Movie 12

Supplemental Movie 13

Supplemental Movie 14

Supplemental Movie 15

Supplemental Movie 16

Supplemental Movie 17

Supplemental Movie 18

Supplemental Movie 19

Supplemental Movie 20

Supplemental Movie 21

Supplemental Movie 22

Supplemental Movie 23

Supplemental Movie 24

## Data Availability

The data that support the findings of this study are available from the corresponding author upon reasonable request.
