# Supplementary material for: Epithelial Cell Mechanoresponse to Matrix Viscoelasticity and Confinement Within Micropatterned Viscoelastic Hydrogels
Source: Adv Sci (Weinh). 2025 Feb 14;12(18):2408635. doi: 10.1002/advs.202408635 (PMC12079340; doi:10.1002/advs.202408635)
Supplement: Supplementary file 1 — Supporting Information [file ADVS-12-2408635-s012.docx]

**Supporting Information for**

**Epithelial Cell Mechanoresponse to Matrix Viscoelasticity and Confinement Within Micropatterned Viscoelastic Hydrogels**

Giuseppe Ciccone^1,2,3^, Mariana Azevedo Gonzalez-Oliva^1,3^, Marie Versaevel^2^, Marco Cantini^3^, Massimo Vassalli^3^, Manuel Salmeron-Sanchez^1,3,4^*and Sylvain Gabriele^2^*

^1^Institute for Bioengineering of Catalonia (IBEC), The Barcelona Institute for Science and Technology (BIST), 08028 Barcelona, Spain

^2^Mechanobiology & Biomaterials Group, University of Mons, Research Institute for Biosciences, CIRMAP, Place du Parc, 20 B-7000 Mons, Belgium

^3^Centre for the Cellular Microenvironment, University of Glasgow, Advanced Research Centre, 11 Chapel Lane, G11 6EW, Glasgow, UK

^4^Institució Catalana de Recerca i Estudis Avançats (ICREA), Barcelona, Spain

*To whom correspondence should be addressed:

[manuel.salmeron-sanchez@glasgow.ac.uk](mailto:manuel.salmeron-sanchez@glasgow.ac.uk); [msalmeron@ibecbarcelona.eu](mailto:msalmeron@ibecbarcelona.eu); [sylvain.gabriele@umons.ac.be](mailto:sylvain.gabriele@umons.ac.be)

**Supporting Note S1. Differences in elasticity values probed by quasi-static nanoindentation and bulk rheology measurements.** The elastic modulus ($G'$*)* measured via bulk rheology **(Supp. Fig. S3)** shows slightly different equivalent absolute values from the Young’s modulus (*E*) measured via nanoindentation **(Fig. 1c).** This discrepancy can be expected due to several reasons, discussed below. First, the fabrication method between hydrogels used for bulk rheology measurements and nanoindentation measurements differs (Experimental Section). Hydrogels used for nanoindentations measurements (and importantly for all cell experiments) are chemically bound to glass coverslips, whereas these used for bulk rheology are not. This changes swelling and other physicochemical properties of hydrogels, which in turn influence their mechanics.^[1]^Therefore we consider the *E* measured by nanoindentation our ground truth value of elasticity. Second, the two measurements entail different length scales (bulk vs microscopic) as well as different deformation modalities (shear vs axial), yielding distinct elastic moduli for synthetic hydrogels, as recently demonstrated in cross-validations studies.^[2]^ Nonetheless, normalisation of results (i.e., dividing $G''$ by $G'$ to obtain tan(*δ*)) yields consistent viscoelasticity measurements to these obtained via nanoindentation stress relaxation experiments **(Fig. 1f).**

**Supporting Table S1.** Hydrogels’ formulations used in this study. E (elastic), V (viscoelastic). OHEA = oxidised N-hydroxyethyl acrylamide, TEMED = tetramethylethylenediamine, APS = ammonium persulfate.

| **Gel name** | **40 % Acrylamide volume (**$\boldsymbol{\mu}$**L)** | **2% Biscrylamide volume (**$\boldsymbol{\mu}$**L)** | **OHEA volume (**$\boldsymbol{\mu}$**L)** | **milli-Q water volume (**$\boldsymbol{\mu}$**L)** | **100% TEMED volume (**$\boldsymbol{\mu}$**L)** | **10% APS volume (**$\boldsymbol{\mu}$**L)** |
| --- | --- | --- | --- | --- | --- | --- |
| Soft E | 100 | 25 | 10 | 855 | 2.5 | 7.5 |
| Soft V | 375 | 5 | 10 | 600 | 2.5 | 7.5 |
| Stiff E | 125 | 50 | 10 | 805 | 2.5 | 7.5 |
| Stiff V | 875 | 6.2 | 10 | 98.8 | 2.5 | 7.5 |

**Supporting Figures**

**
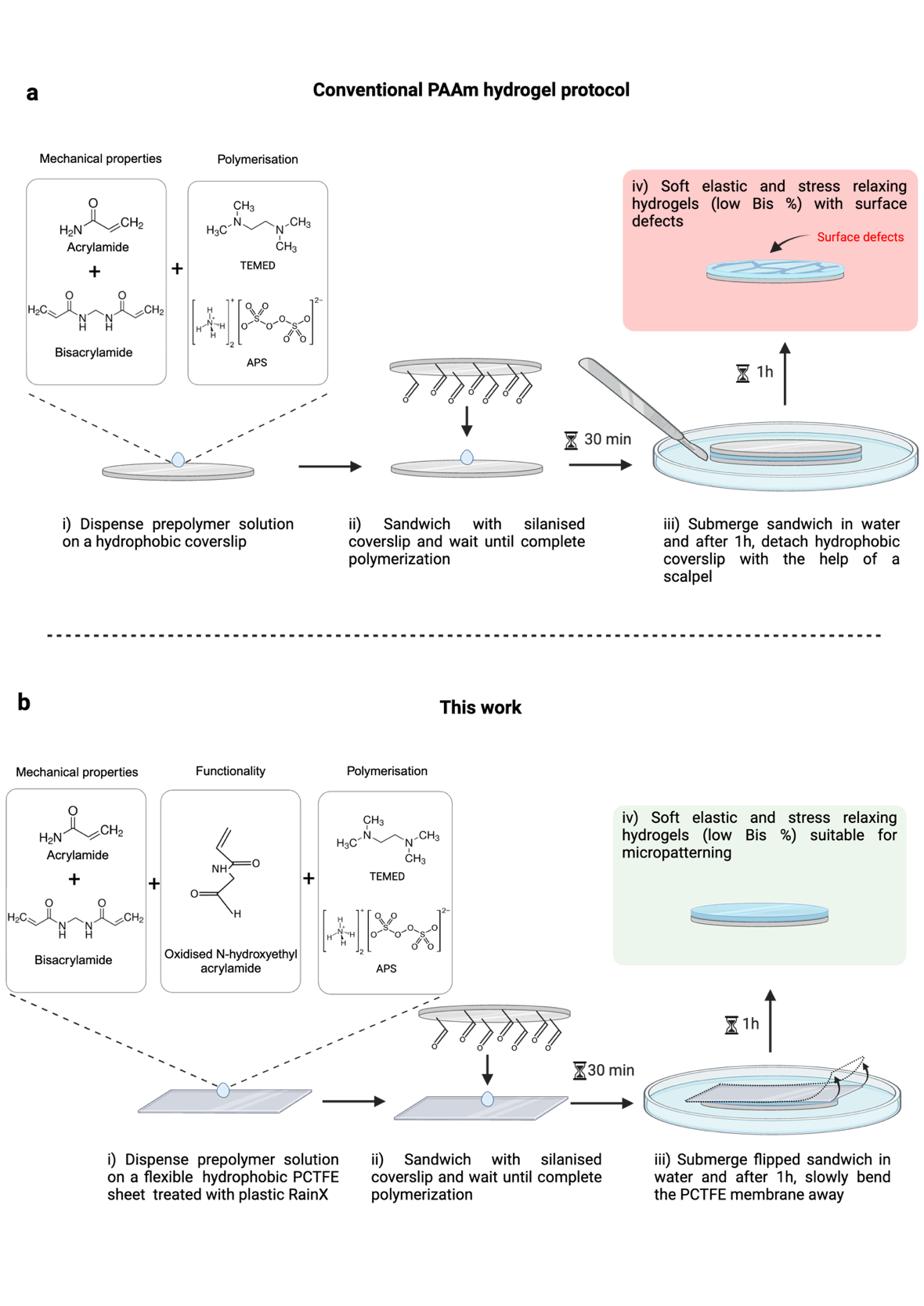
**

**Supporting Figure S1. Manufacturing processes of elastic and viscoelastic PAAm hydrogels. (a)** Conventional fabrication workflow of elastic PAAm hydrogels of varying stiffnesses^[3]^ yields surface defects in hydrogels with low Bis % (soft E and stress relaxing hydrogels). **(b)** Workflow developed in this manuscript to fabricate soft and viscoelastic PAAm hydrogels of varying mechanical properties and suitable for micropatterning. This involves polymerising hydrogels against a flexible, oxygen-impermeable Polychlorotrifluoroethylene (PCTFE) support treated with plastic Rain X instead of an infinitely stiff glass substrate. Created in <https://BioRender.com>.


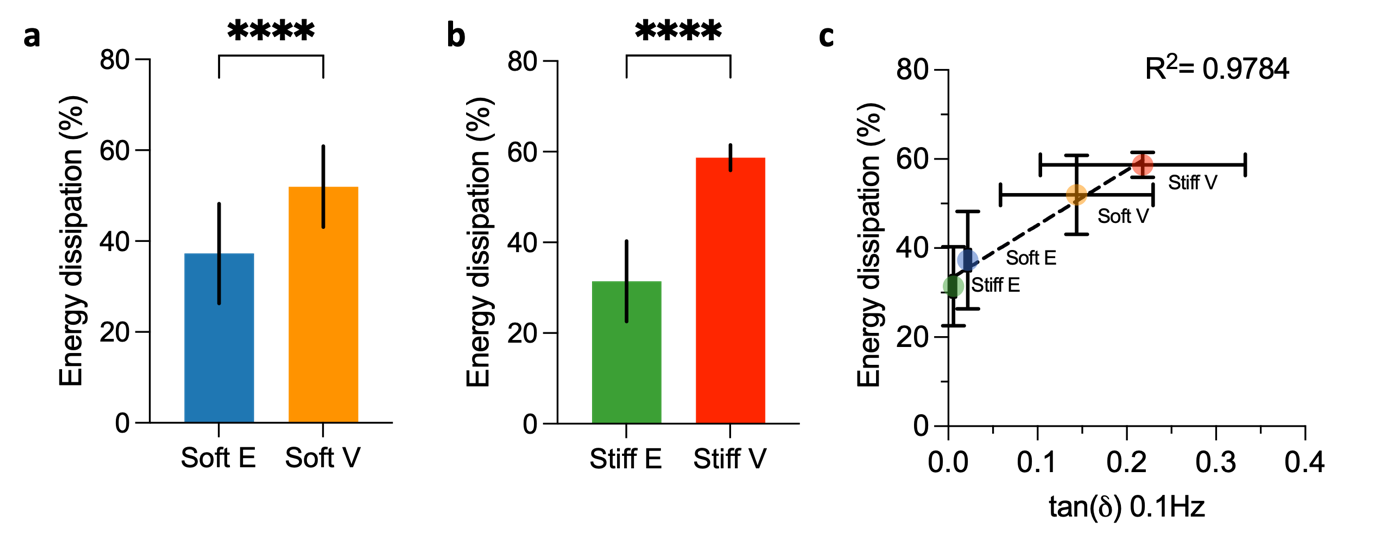


**Supporting Figure S2 – Viscoelastic properties of soft and stiff polyacrylamide hydrogels. (a)** Average energy dissipation over $\sim$ 60 s for soft elastic (Soft E) and viscoelastic (Soft V) hydrogels. Data is shown as mean $\pm$ SD (n = 131 for Soft E, n = 151 for soft V over at least two independent samples). **(b)** Average energy dissipation over $\sim$ 60 s for stiff elastic (Stiff E) and viscoelastic (Stiff V) hydrogels. Data is shown as mean $\pm$ SD (n = 142 for stiff E, n = 121 for stiff V over at least two independent samples). **(c)** Average energy dissipation over $\sim$ 60 plotted against the tan($\delta$) at 0.1 Hz. Data is shown as mean $\pm$ SD, where the number of points for the relaxation half time is the same as in a-b, and the number of independent samples for the tan($\delta$) is 3 (R^2^=0.9784).


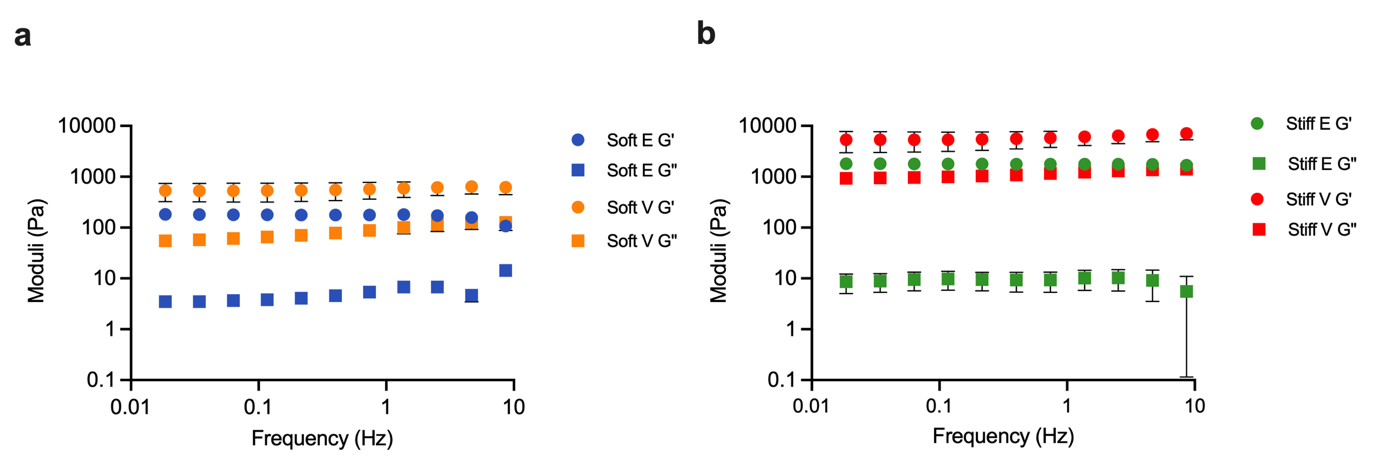


**Supporting Figure S3.** $G'$and $G''$ obtained from bulk rheology experiments (strain 1%, Experimental Section). **(a)** Soft hydrogels group. **(b)** Stiff hydrogels group. Data is shown as mean $\pm$ SD from three independent hydrogels per condition.


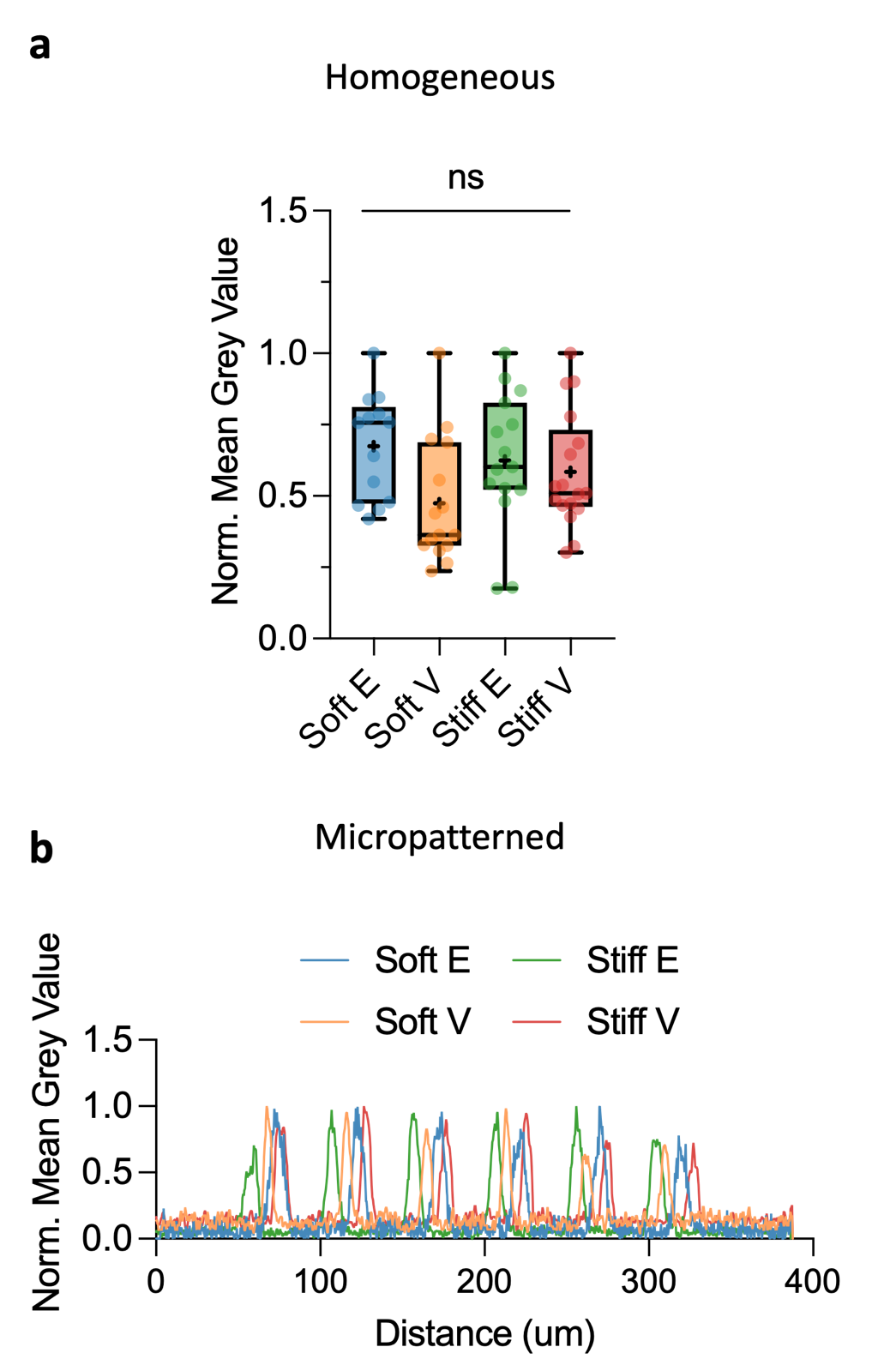


**Supporting Figure S4. (a)** Normalised Mean Grey Value (Norm. Mean Grey Value) of fluorescently labelled fibronectin covalently linked to hydrogels developed in this work. Each data point represents a random area in the central region of the hydrogel, n=3 hydrogels per condition. ns p>0.05, two-way ANOVA with Tukey’s multiple comparisons test. **(b)** Normalised Mean Grey Value (Norm. Mean Grey Value) of covalently stamped fibronectin on the hydrogels developed in this work. Peaks show comparable levels of micropattern fibronectin on each hydrogel, consistent with data in (a).

**
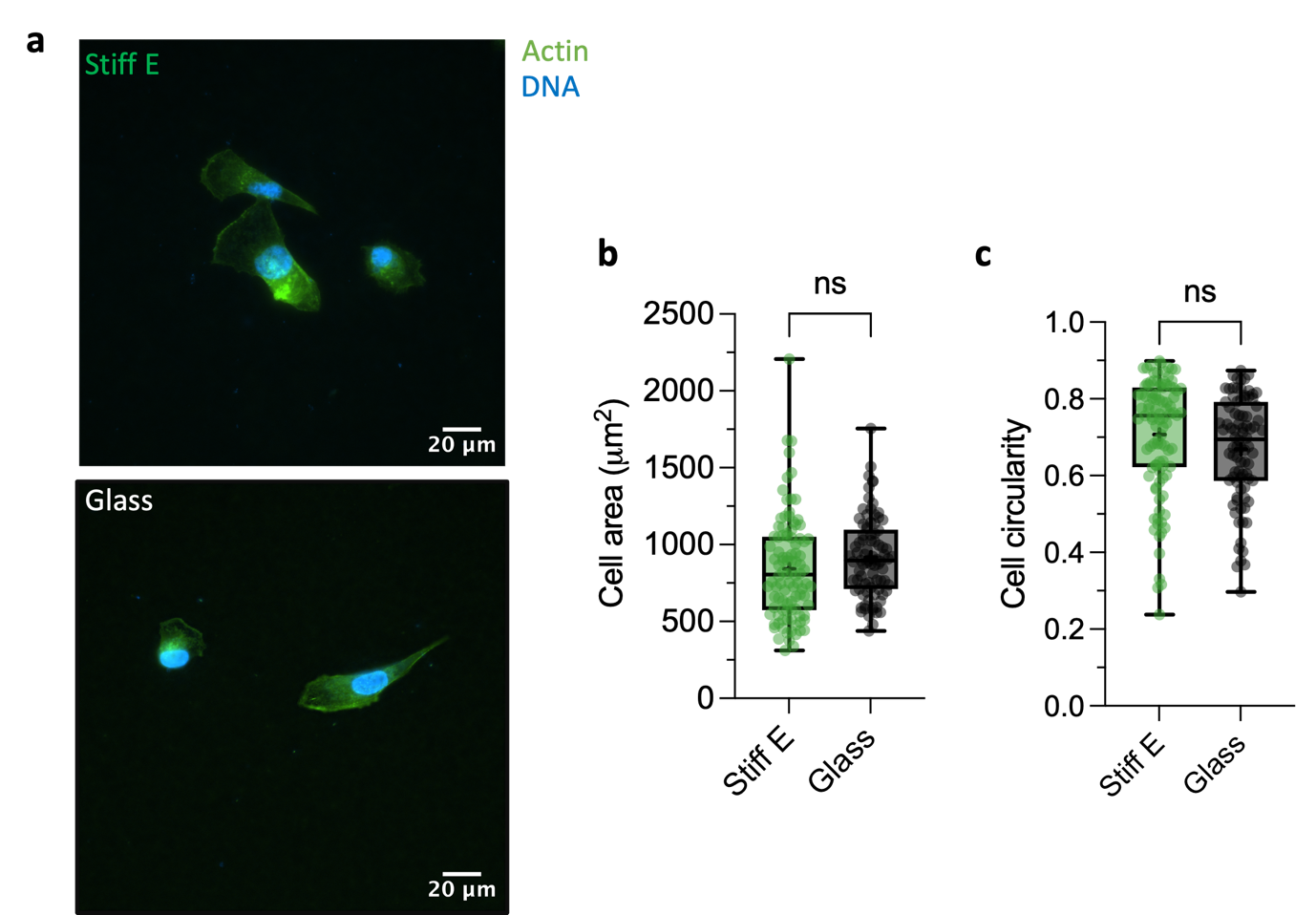
**

**Supporting Figure S5 – Cell spreading area and circularity are the same on stiff elastic (Stiff E) hydrogels and fibronectin coated glass coverslips (Glass). (a)** Representative Actin/DNA images of MCF-10A cells cultured on stiff elastic hydrogels (Stiff E) and fibronectin-coated glass coverslips. **(b)** Quantification of MCF-10A cell spreading area on stiff elastic (Stiff E) hydrogels (n = 100 cells) and fibronectin-coated glass coverslips (Glass) (n = 86 cells) from at least two independent experiments. ns p = 0.0985, two-tailed unpaired t-test. **(c)** Quantification of MCF-10A cell circularity on stiff elastic (Stiff E) hydrogels (n = 100 cells) and fibronectin-coated glass coverslips (Glass) (n = 86 cells) from at least two independent experiments. ns p = 0.0810, two-tailed unpaired t-test.


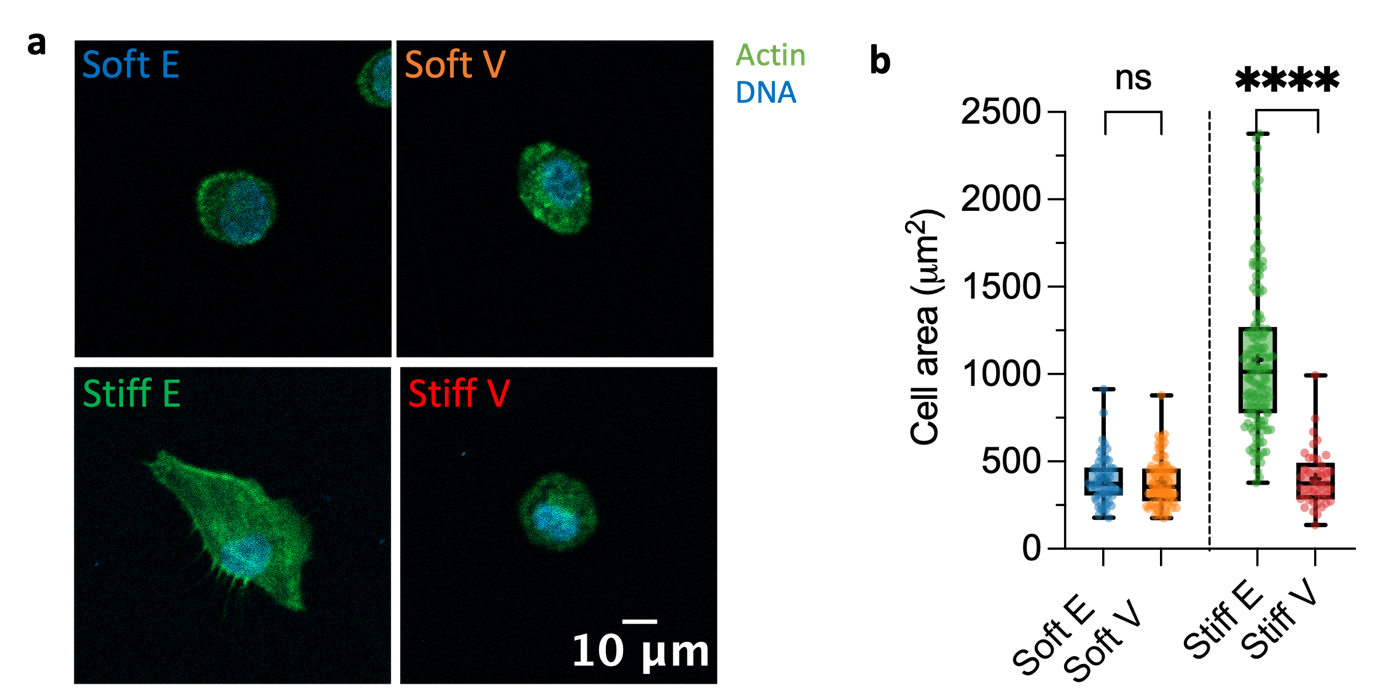


**Supporting Figure S6 – Cell spreading on elastic and viscoelastic polyacrylamide hydrogels coated with low fibronectin concentration. (a)** Representative Actin/DNA images of MCF-10A cells cultured on viscoelastic polyacrylamide hydrogels coated with low fibronectin concentration. **(b)** Quantification of the cell spreading area of MCF-10A cells cultured on viscoelastic polyacrylamide hydrogels coated with low fibronectin concentration (n= 61 cells for Soft E, n = 84 cells for Soft V, n = 138 cells for Stiff E, n = 41 cells for Stiff V from two independent experiments). ns p >0.9999, ****p<0.0001, two-way ANOVA with Bonferroni’s multiple comparisons test.


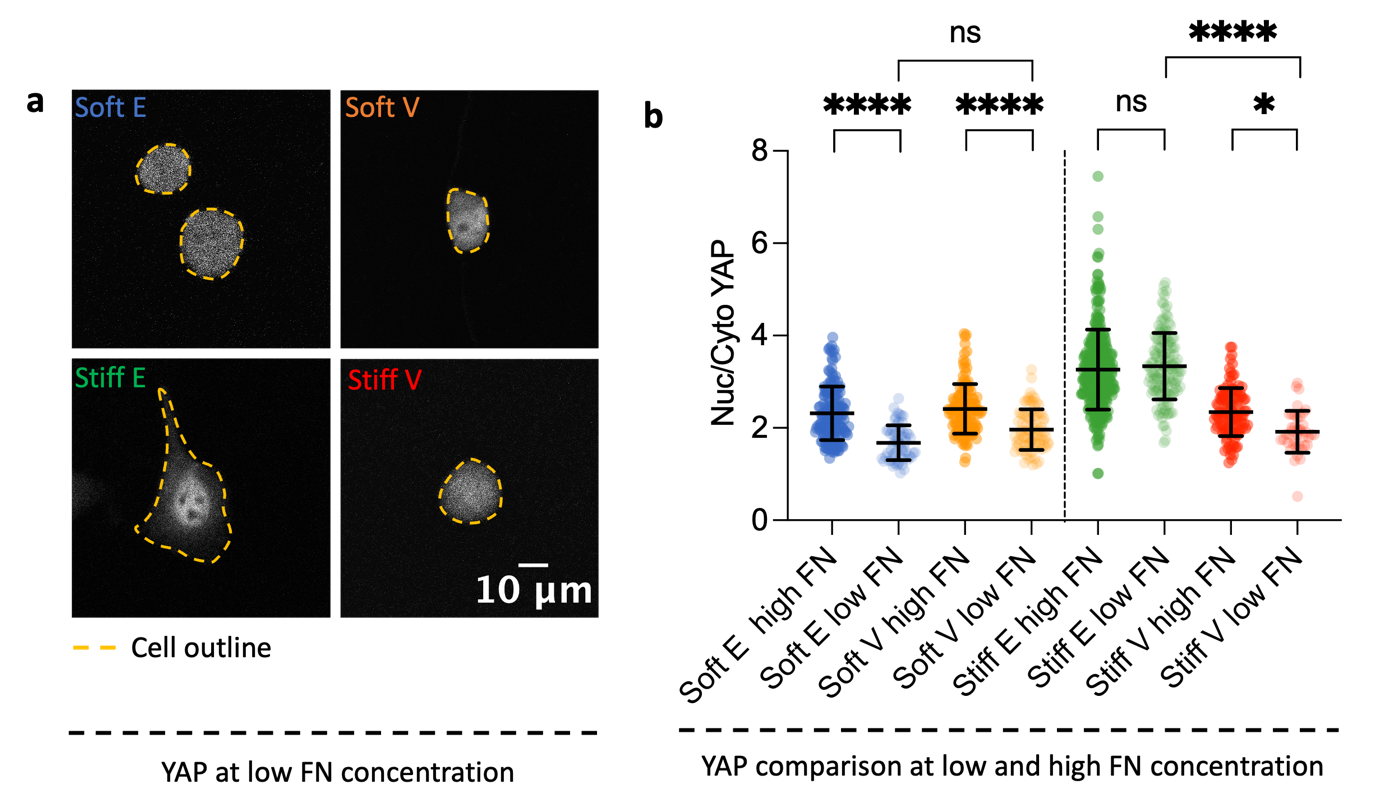


**Supporting Figure S7 – YAP nuclear translocation on elastic and viscoelastic polyacrylamide hydrogels coated with low fibronectin (FN) concentration.** (a) Representative fluorescence images of MCF-10A cells cultured on viscoelastic polyacrylamide hydrogels coated with low FN concentration and stained for YAP. The cellular outline is depicted by a dashed yellow line. **(b)** Quantification of the Nuclear to Cytoplasmic (Nuc/Cyto) YAP ratio of MCF-10A cells cultured on viscoelastic polyacrylamide hydrogels coated with high and low FN concentration. Data for high FN concentration is presented in Fig. 2. Data for low FN concentration is as follows: n= 61 cells for Soft E, n = 84 cells for Soft V, n = 138 cells for Stiff E, and n = 41 cells for Stiff V from two independent experiments. Data is shown as mean $\pm$ SD. ns p > 0.05, * p < 0.0107, **** p< 0.0001, three-way ANOVA with Tukey’s multiple comparisons test.

**Supporting Figure S8 – Evolution of the Nuclear to Cytoplasmic YAP ratio (Nuc/Cyto) YAP as a function of the number of focal adhesions per cell (# FAs/cell)** **on elastic and viscoelastic polyacrylamide hydrogels.** Data is shown as mean $\pm$ SEM. For YAP, sample size is as follows: soft E: n=157 cells, Soft V: n=128 cells, Stiff E: n=320 cells, and Stiff V: n=113 cells, from at least two independent experiments. For FAs, sample size is as follows: soft E: n = 33 cells, soft V: n=41 cells, Stiff E: n=58 cells, and stiff V: n=27 cells, from at least two independent experiments.

**
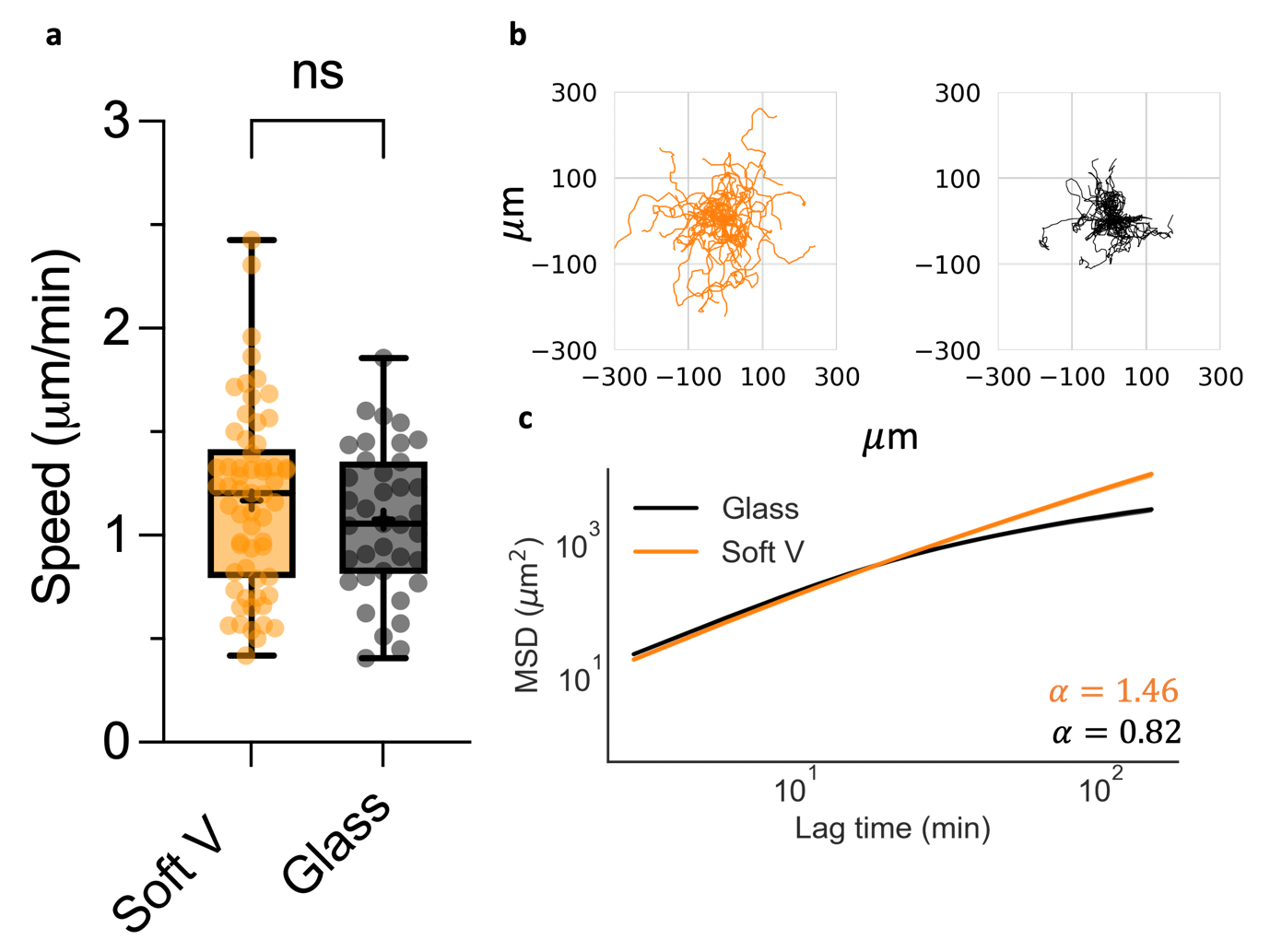
**

**Supporting Figure S9 – MCF-10A cells exhibit comparable migration speed but higher directional persistence on soft viscoelastic (Soft V) matrices compared to fibronectin-coated glass coverslips (Glass). (a)** Quantification of MCF-10A cell migration speed (n= 61 cells for Soft V, n = 37 cells for Glass) from at least two independent experiments. ns p = 0.2842, two-tailed unpaired t-test. **(b)** Representative trajectories of MCF-10A cells migration on soft V and glass (n=43 cells for Soft V, n= 33 cells for Glass) from at least two independent experiments. **(c)** Average mean square displacement (MSD) vs lag-time for MCF-10A cells on Soft V and Glass from at least two independent experiments. The diffusion exponent, $\alpha$*,* is shown in the graph. Data is shown as mean $\pm$ SEM (n=43 cells for Soft V, n= 33 cells for Glass).


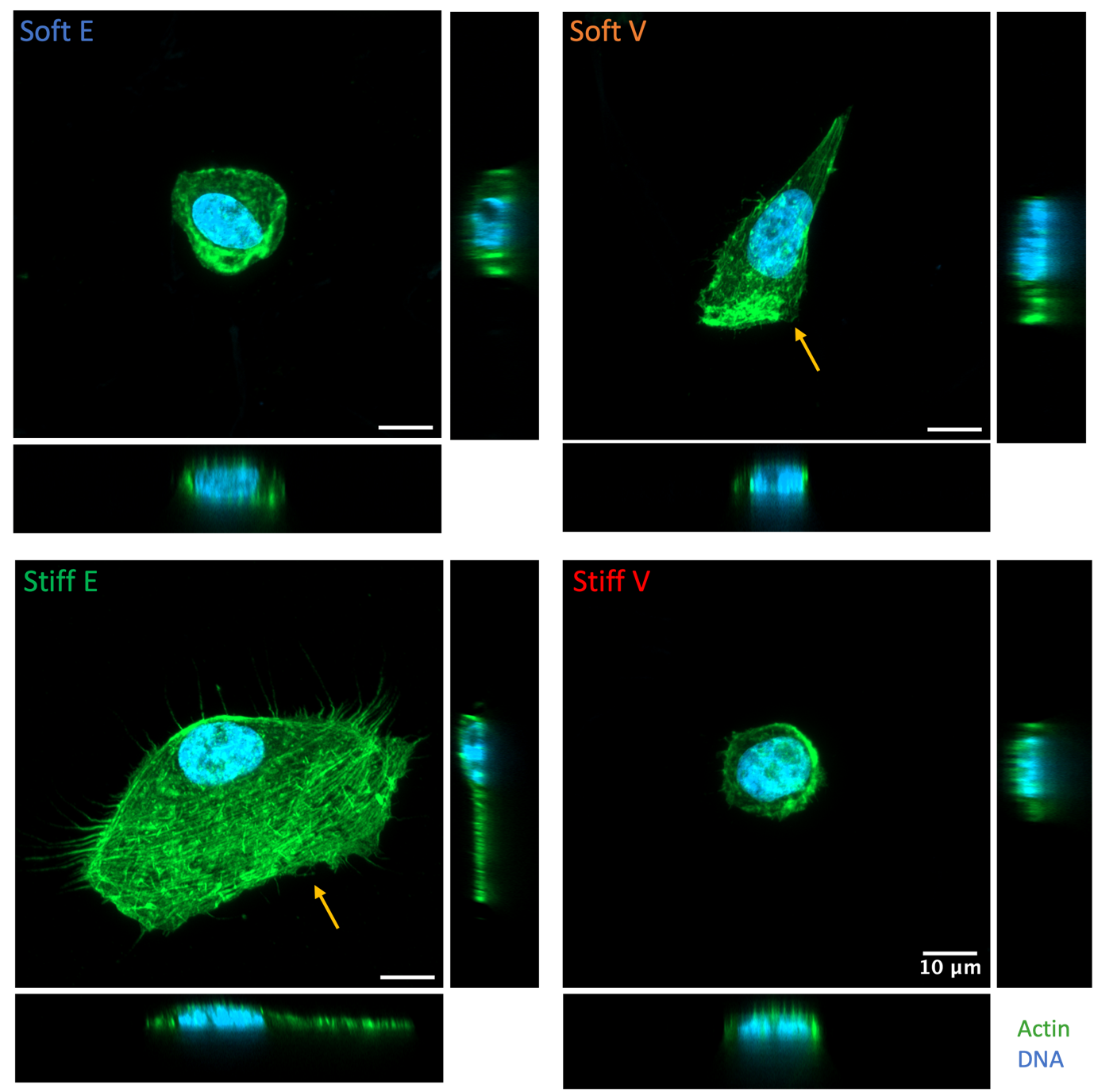


**Supporting Figure S10 – Lamellipodium formation on elastic and viscoelastic polyacrylamide hydrogels.** Representative maximum intensity projections of MCF-10A cells cultured on elastic and viscoelastic polyacrylamide hydrogels. Corresponding (*xz*) projections (horizontal) and (*yz*) projections (vertical) are shown alongside each image. Lamellipodium is indicated by a yellow arrow. Images for soft E and stiff V are the same as in Fig. 1a. All scale bars are 10 μm.


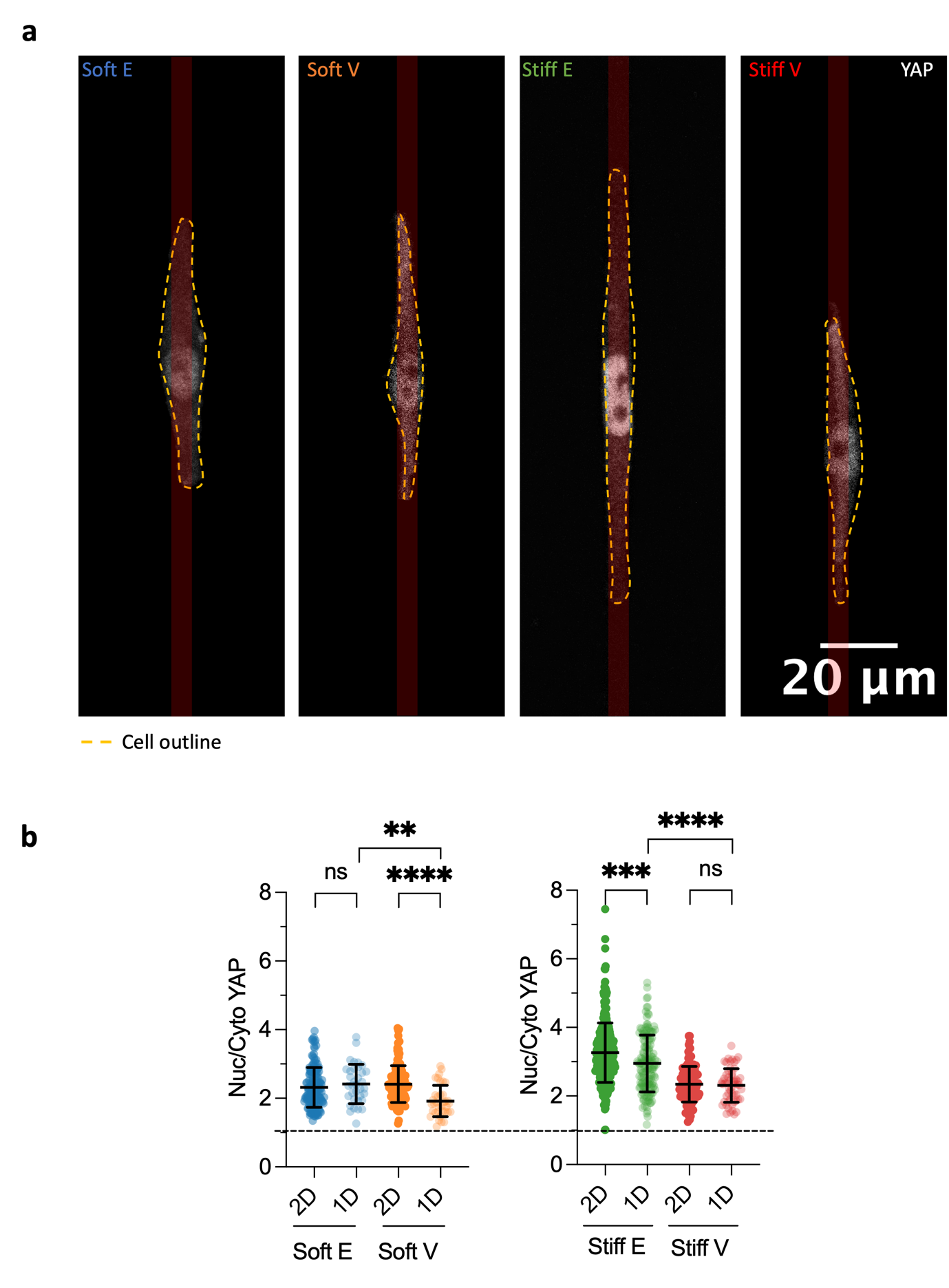


**Supporting Figure S11 – YAP nuclear translocation of MCF-10A cells on 5 μm micropatterned fibronectin lines on elastic and viscoelastic polyacrylamide hydrogels.** **(a)** Representative fluorescence images of MCF-10A cells cultured on micropatterned viscoelastic polyacrylamide hydrogels and stained for YAP. The cellular outline is depicted by a dashed yellow line, and fibronectin micropatterns are schematically represented in red. **(b)** Quantification of nuclear to cytoplasmic (Nuc/Cyto) YAP ratio on 2D and 1D soft elastic (Soft E) and viscoelastic (Soft V) matrices, left graph (n = 157 cells for Soft E 2D, n = 35 cells for soft E 1D, n = 128 cells for Soft V 2D, n = 36 cells for soft V 1D) from at least two independent experiments. Dashed line indicates a Nuc/Cyto YAP ratio of 1. ****p < 0.0001, **p = 0.001, two-way ANOVA with Tukey’s multiple comparisons test. Quantification of nuclear to cytoplasmic (Nuc/Cyto) YAP ratio on 2D and 1D stiff elastic (Stiff E) and viscoelastic (Stiff V) matrices, right graph (n=320 cells for Stiff E 2D, n = 150 cells for stiff E 1D, n=113 cells for stiff V 2D, n = 56 cells for stiff V 1D) from at least two independent experiments. ****p<0.0001, ***p=0.0003, ns p = 0.9911, two-way ANOVA with Tukey’s multiple comparisons test.


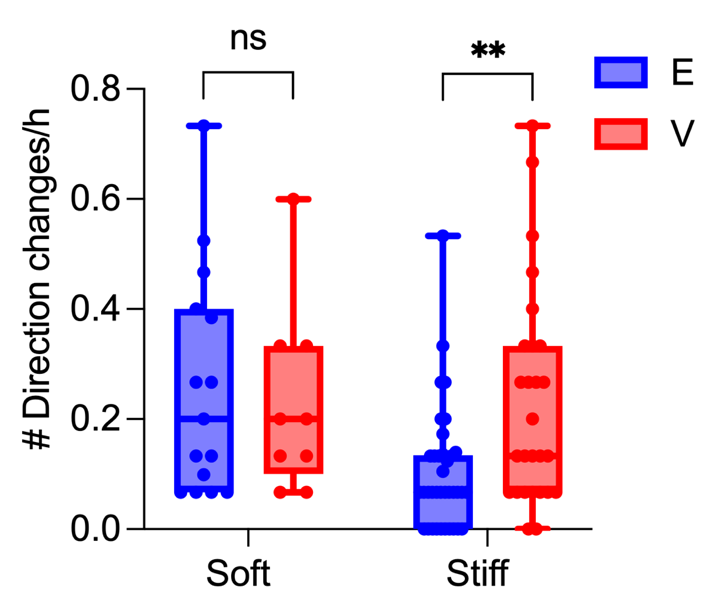


**Supporting Figure S12. Number of directions changes per hour (# Direction changes/h) of MCF-10A confined to 1D fibronectin lines over a period of 15 hours.** Each dot represents a single cell (n=15 Soft E, n= 9 Soft V, n=25 Stiff E, n=22 Stiff V) from at least two independent experiments per condition. ns p>0.05, **p=0.006, two-way ANOVA with Šídák multiple comparisons test.

**Supporting Movies**

**Supporting Movie S1 –** Time lapse movie in DIC mode of MCF-10A cells migrating for 15h on a stiff elastic (Stiff E) hydrogel substrate.

**Supporting Movie S2 –** Time lapse movie in DIC mode of MCF-10A cells migrating for 15h on a stiff viscoelastic (Stiff V) hydrogel substrate.

**Supporting Movie S3 –** Time lapse movie in DIC mode of MCF-10A cells migrating for 15h on a soft elastic (Soft E) hydrogel substrate.

**Supporting Movie S4 –** Time lapse movie in DIC mode of MCF-10A cells migrating for 15h on a soft viscoelastic (Soft V) hydrogel substrate.

**Supporting Movie S5 –** Confocal volume rendering of a single MCF-10A cell on a stiff elastic (Stiff E) hydrogel substrate stained for actin (green) and DNA (blue).

**Supporting Movie S6 –** Confocal volume rendering of a single MCF-10A cell on a stiff viscoelastic (Stiff V) hydrogel substrate stained for actin (green) and DNA (blue).

**Supporting Movie S7 –** Confocal volume rendering of a single MCF-10A cell on a soft elastic (Soft E) hydrogel substrate stained for actin (green) and DNA (blue).

**Supporting Movie S8 –** Confocal volume rendering of a single MCF-10A cell on a soft viscoelastic (Soft V) hydrogel substrate stained for actin (green) and DNA (blue).

**Supporting Movie S9 –** Time lapse movie in confocal fluorescence mode of a single MCF-10A tagged with Spy-555-FastAct on a stiff elastic (Stiff E) hydrogel substrate.

**Supporting Movie S10 –** Time lapse movie in confocal fluorescence mode of a single MCF-10A tagged with Spy-555-FastAct on a stiff viscoelastic (Stiff V) hydrogel substrate.

**Supporting Movie S11 –** Time lapse movie in confocal fluorescence mode of a single MCF-10A cell tagged with Spy-555-FastAct on a soft elastic (Soft E) hydrogel substrate.

**Supporting Movie S12 –** Time lapse movie in confocal fluorescence mode of a single MCF-10A cell tagged with Spy-555-FastAct on a soft viscoelastic (Soft V) hydrogel substrate.

**Supporting Movie S13 –** Time lapse movie of a single MCF-10A cell transfected with CellLight™ Talin-GFP, BacMam 2.0 on a soft elastic (Soft E) hydrogel substrate.

**Supporting Movie S14 –** Time lapse movie of a single MCF-10A cell transfected with CellLight™ Talin-GFP, BacMam 2.0 on a soft viscoelastic (Soft V) hydrogel substrate.

**Supporting Movie S15 –** Time lapse movie of a single MCF-10A cell transfected with CellLight™ Talin-GFP, BacMam 2.0 on a stiff elastic (Stiff E) hydrogel substrate.

**Supporting Movie S16 –** Time lapse movie of a single MCF-10A cell transfected with CellLight™ Talin-GFP, BacMam 2.0 on a stiff viscoelastic (Stiff V) hydrogel substrate.

**Supporting Movie S17 –** Time lapse movie of a single MCF-10A cell transfected with CellLight™ Talin-GFP, BacMam 2.0 on a soft elastic (Soft E) micropatterned hydrogel substrate.

**Supporting Movie S18 –** Time lapse movie of a single MCF-10A cell transfected with CellLight™ Talin-GFP, BacMam 2.0 on a soft viscoelastic (Soft V) micropatterned hydrogel substrate.

**Supporting Movie S19 –** Time lapse movie of a single MCF-10A cell transfected with CellLight™ Talin-GFP, BacMam 2.0 on a stiff elastic (Stiff E) micropatterned hydrogel substrate.

**Supporting Movie S20 –** Time lapse movie of a single MCF-10A cell transfected with CellLight™ Talin-GFP, BacMam 2.0 on a stiff viscoelastic (Stiff V) micropatterned hydrogel substrate.

**Supporting Movie S21–** Time lapse movie of MCF-10A cells migrating for 15h on a stiff elastic (Stiff E) micropatterned hydrogel substrate with 5 μm fibronectin lines (DIC mode in grey and DNA in blue).

**Supporting Movie S22 –** Time lapse movie of MCF-10A cells migrating for 15h on a stiff viscoelastic (Stiff V) micropatterned hydrogel substrate with 5 μm fibronectin lines (DIC mode in grey and DNA in blue).

**Supporting Movie S23 –** Time lapse movie of MCF-10A cells migrating for 15h on a soft elastic (Soft E) micropatterned hydrogel substrate with 5 μm fibronectin lines (DIC mode in grey and DNA in blue).

**Supporting Movie S24 –** Time lapse movie of MCF-10A cells migrating for 15h on a soft viscoelastic (Soft V) micropatterned hydrogel substrate with 5 μm fibronectin lines (DIC mode in grey and DNA in blue).

**Supporting References**

[1] R. Subramani, A. Izquierdo-Alvarez, P. Bhattacharya, M. Meerts, P. Moldenaers, H. Ramon, H. Van Oosterwyck, *Frontiers in Materials* **2020**, *7*.

[2] N. R. Richbourg, M. K. Rausch, N. A. Peppas, *Polymer* **2022**, *258*, 125316.

[3] J. R. Tse, A. J. Engler, *Current Protocols in Cell Biology* **2010**, *47*, 1.
